# Extremely halophilic brine community manipulation shows higher robustness of microbiomes inhabiting human-driven solar saltern than naturally driven lake

**DOI:** 10.1128/msystems.00538-24

**Published:** 2024-06-27

**Authors:** Raquel Liébana, Tomeu Viver, María Dolores Ramos-Barbero, Esteban Bustos-Caparros, Mercedes Urdiain, Cristina López, Mohammad Ali Amoozegar, Josefa Antón, Ramon Rossello-Mora

**Affiliations:** 1 Marine Microbiology Group, Department of Animal and Microbial Biodiversity, Mediterranean Institute for Advanced Studies (IMEDEA, UIB-CSIC), Esporles, Spain; 2 Department of Molecular Ecology, Max Planck Institute for Marine Microbiology, Bremen, Germany; 3 Department of Physiology, Genetics and Microbiology, University of Alicante, Alicante, Spain; 4 Department of Genetics, Microbiology and Statistics, University of Barcelona, Barcelona, Spain; 5 Extremophiles Laboratory, Department of Microbiology, School of Biology and Center of Excellence in Phylogeny of Living Organisms, College of Science, University of Tehran, Tehran, Iran; University of California San Diego, La Jolla, California, USA

**Keywords:** community assembly, microdiversity, comparative metagenomics, hyperhalophilic microbiomes, community transplant, viral predation pressure, metagenome-assembled genomes

## Abstract

Hypersaline ecosystems display taxonomically similar assemblages with low diversities and highly dense accompanying viromes. The ecological implications of viral infection on natural microbial populations remain poorly understood, especially at finer scales of diversity. Here, we sought to investigate the influence of changes in environmental physicochemical conditions and viral predation pressure by autochthonous and allochthonous viruses on host dynamics. For this purpose, we transplanted two microbiomes coming from distant hypersaline systems (solar salterns of Es Trenc in Spain and the thalassohaline lake of Aran-Bidgol lake in Iran), by exchanging the cellular fractions with the sterile-filtered accompanying brines with and without the free extracellular virus fraction. The midterm exposure (1 month) of the microbiomes to the new conditions showed that at the supraspecific taxonomic range, the assemblies from the solar saltern brine more strongly resisted the environmental changes and viral predation than that of the lake. The metagenome-assembled genomes (MAGs) analysis revealed an intraspecific transition at the ecotype level, mainly driven by changes in viral predation pressure, by both autochthonous and allochthonous viruses.

**IMPORTANCE:**

Viruses greatly influence succession and diversification of their hosts, yet the effects of viral infection on the ecological dynamics of natural microbial populations remain poorly understood, especially at finer scales of diversity. By manipulating the viral predation pressure by autochthonous and allochthonous viruses, we uncovered potential phage–host interaction, and their important role in structuring the prokaryote community at an ecotype level.

## INTRODUCTION

Unravelling the ecological mechanisms influencing biological diversity is still a major challenge in community ecology (1). The high microdiversity found in metagenomic surveys reveals that the community assembly processes may be more complex than that observed at a coarser diversity scale. There is no agreement on why this microdiversity coexists spatially and persists in time, and whether it is phenotypically relevant (2). Some studies suggest that a weak intraspecies competition results in genetic drift, which in turn maintains or promotes microdiversity within taxa (3). However, it has been reported that microdiversity within species has functional consequences, which lead to niche partitioning, providing stability under unfavorable conditions and sustaining the persistence of those species (4–6). This has been reported for dominant and persistent taxa inhabiting marine (i.e., *Prochlorococcus* [7, 8]) and freshwater (i.e., *Polynucleobacter asymbioticus* [9]), soil (i.e., *Curtobacterium* [10]) or hypersaline habitats like solar saltern brines, where we have recently shown the coexistence of different ecotypes within the dominant archaea *Haloquadratum walsbyi* (11) and bacteria *Salinibacter ruber* (12), displaying different dynamics as response to salinity changes.

Microdiversity is originated by genetic variation through horizontal gene transfer and mutation. This genetic diversity is higher in the flexible genome of a given species, subjected to a high turnover, than in the core genome shared by the different subpopulations or ecotypes within a species. The flexible genome is often clustered into genomic islands enriched by insertion sites for mobile genetic elements, such as transposable elements or temperate phages (13, 14). It has been observed that mobile genetic elements and genomic islands often encode genes involved in cell surface component synthesis and defense mechanisms such as clustered regularly interspaced short palindromic repeats (CRISPR) systems. Therefore, this evidences the central role of species–species and virus–host interactions in generating and maintaining microdiversity. Particularly, the coevolution of viruses and their hosts drives diversity within subpopulations or ecotypes (15, 16). Additionally, it has been postulated that the coexistence of subpopulations results from a negative frequency-dependent selection, discussed by the “killing the winner” (KtW) hypothesis (17) and further developed by the “constant diversity” (CD) hypothesis including metagenomic data (18).

Hypersaline aquatic ecosystems offer an excellent opportunity to assess the importance of different ecological processes during community assembly at fine diversity scales. These habitats are discrete and globally scattered, where salinity is a major driver of community assembly, selecting for similar microbial halophilic assemblages. At salt-saturated conditions, hypersaline communities typically harbor high densities of cells (~10^8^ cell mL^−1^) and virus-like particles (VLPs), up to 10^10^ VLP mL^−1^, the highest among aquatic ecosystems (16, 19). Brines host a relatively reduced prokaryotic taxonomic diversity, mainly dominated by representatives of the archaeal classes *Halobacteria* and *Nanohaloarchaea* and by members of the bacterial family of *Salinibacteraceae* (20). This reduced richness in species and higher taxa represent an advantage for metagenomic studies as we can bin metagenome-assembled genomes (MAGs) of the dominating taxa. One of the benefits of working with MAGs is that they represent the mosaic of all major populations within a species (21), and therefore the changes in the gene composition should generally reflect changes in genome abundances of single populations, allowing the raw measures of the intraspecies diversity. Indeed, the analysis of genomes and MAGs has revealed halophilic microbiomes to possess a high microdiversity. Commonly dominating taxa such as *Hqr. walsbyi* (22) and *Sal. ruber* (12, 23, 24) display high intraspecies diversity.

In the current study, we have challenged two different microbial communities from hypersaline environments of distant origin and distinct environmental conditions by permutating their cell biomasses, viruses, and filtered brines to observe how they interact and react at the short temporal scale. With this setup, we asked (i) what is the role of (micro)diversity in species stability and persistence and, ultimately, community response to environmental changes; (ii) what is the influence of virus–host interactions in structuring gene content and turnover among subpopulations; and (iii) are allochthonous viruses able to infect cells from an alien community assemblage, and if so, does the infection pressure cause similar effects in the community as those caused by autochthonous viruses? Aiming to address these questions, we have first “deconstructed” the community assemblages, to reconstruct them by interchanging their cellular and viral assemblages, as well as the cell and virus-free tangentially filtered brines. To assess if changes in the viral predation pressure affected the community assembly process, we removed the viral suspended fraction, keeping only those viruses that were already infecting or adsorbed to the cells. Duplicated microcosms were used for comparative metagenomics to assess whether the change of environment and/or in viral predation pressure by autochthonous/allochthonous viruses in brines generated an ecological response at the ecotype level in the transplanted communities. We hypothesized that, as observed at higher taxonomic ranks, a high microdiversity in taxa would ensure their persistence under changes in the environmental conditions.

## RESULTS

### Transplantation of cells, brines, and the accompanying virosphere

**Fig 1:**
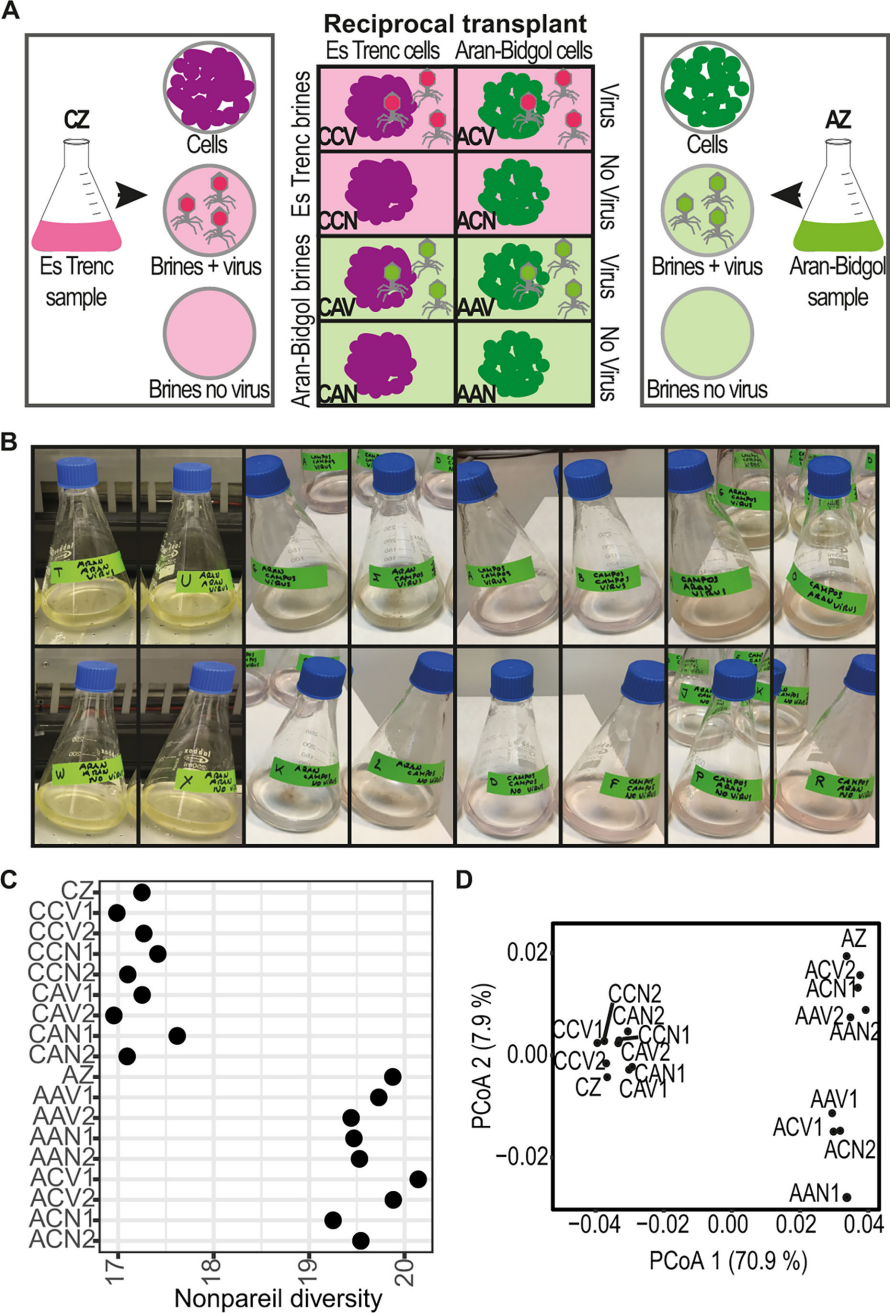
(**A**) Graphical representation of the experimental setup; (**B**) microcosms at the end of the experiment after 33 days; (**C**) Nonpareil diversity of metagenomes; (**D**) principal coordinate analysis (PCoA) plot based on MASH-based distance among metagenome reads. Sample name nomenclature is as follows: the first letter refers to cell origin (C, Es Trenc; A, Aran-Bidgol), the second to brine origin (C, Es Trenc; A, Aran-Bidgol), and the third to the presence/absence of the suspended brine viral fraction in the given brine (V, presence of viruses; N, absence of viruses). AZ and CZ refer to Aran-Bidgol and Es Trenc inocula, respectively.

This study was conducted with brines from two distant hypersaline habitats: (i) a crystallizer pond from Es Trenc solar saltern (Campos, Mallorca, Spain), which has been used for salt harvest for decades, and (ii) the Aran-Bidgol thalasohaline lake (central Iran), which reaches salt saturation during summer when the lake is nearly dry. The former can be considered as a human-driven semiartificial system with controlled regular cycles of filling and evaporation, whereas the latter is a naturally occurring environment with little human influence and exposed only to the natural climate changes. We reciprocally transplanted the cellular fractions from each site to either the filter-sterilized virus-free brine, or to the virus-containing brine from the other site (Fig. 1A; Fig. S1). The inoculum brines collected from the solar saltern and the lake had a salinity of 35.6% and 31.6%, respectively, at the time of sampling, with Na^+^, Mg^2+^, Cl^-^ y SO_4_^2-^ as the main ions (Table S1). Both inoculum samples displayed similar cell concentrations and a dominance of archaea over bacteria: Es Trenc 8.3 × 10^6^ cells mL^−1^ for archaea and 2.6 × 10^6^ cells mL^−1^ for bacteria, and Aran-Bidgol 4.8 × 10^6^ cells mL^−1^ for archaea and 2.6 × 10^6^ cells mL^−1^ for bacteria. Both also displayed similar number of viruses, with around 6 × 10^9^ VLP mL^−1^ (6.85 × 10^9^ ± 7.42 × 10^7^ and 5.71 × 10^9^ ± 1.15 × 10^8^ in Es Trenc and Aran-Bidgol, respectively), with head-tailed, spherical, filamentous, baciliform, bottle-shaped, pleomorphic, and spindle-shaped morphotypes (Fig. S2). Differences in the color of brines were observed at the end of the experiment; pink-red for those containing cells from Es Trenc and green for cells from Aran-Bidgol (Fig. 1B). Both archaea and bacteria increased in numbers during the experiment in all microcosms (Fig. S3), with bacteria to archaea ratios showing inverted trends of 0.27 and 0.49 at the beginning of the experiment, and 0.72 and 0.08 at the end of the experiment in the cellular biomasses of Es Trenc and Aran-Bidgol, respectively, with independence of the extracellular environment.

### Higher stability displayed by the solar saltern microbial community than that of the lake

Metagenome characteristics of the inoculum brine samples from Es Trenc solar saltern and Aran-Bidgol lake and of the 16 microcosms at the end of the experiment are provided in Table S2. The experimental manipulation of the communities (i.e., reciprocal cell and suspended viral fraction transplant that we consider an “environment exchange”) did not cause a drop in community alpha-diversity as estimated by the Nonpareil sequence diversity (Fig. 1C). Samples with cells from Aran-Bidgol were always more diverse than that from Es Trenc (19.53 ± 0.56 and 17.18 ± 0.35, respectively). Relatedness among metagenomic reads, calculated with MASH distance (Table S3), indicated that the environment exchange caused metagenomes from Aran-Bidgol to display a higher dispersion (0.03–0.05) than those of Es Trenc (0.02–0.03) after 1 month of incubation (Fig. 1D). The increase in community dissimilarity along the experiments in the Aran-Bidgol samples was generalized, regardless of the experimental manipulation, observing a high dispersion even in microcosms where the original environment was maintained (i.e., AZ, AAV1, and AAV2). Additionally, samples from Aran-Bidgol were grouped into two clusters, with samples AAV1, ACV1, ACN2, and AAN1 markedly separated from samples clustering with the inoculum (mean MASH distance from AZ of 0.048, mean intracluster distance of 0.038).

A total of 89 MAGs were recovered, 66 and 23 from Es Trenc and Aran-Bidgol samples, respectively (Table 1; Table S4). According to their reciprocal ANI (>95%), the binned MAGs conformed a total of 18 genomospecies in Es Trenc and 14 in Aran-Bidgol. Only three genomospecies were shared between Es Trenc and Aran-Bidgol (Table S5), which affiliated with *Sal. ruber* (reciprocal ANI 97.87–99.80), *Hqr. walsbyi* (reciprocal ANI 99.47–99.87), and *Halonotius* sp. (reciprocal ANI 98.90). Representative genomospecies MAGs were chosen according to their quality, with a completeness from 70% to 90% in 24 MAGs, and over 90% in 14 (Table S4), which were used for downstream analysis. All genomospecies retrieved from Aran-Bidgol (representative MAGs) accounted for 34%–48% of the total microbial population, whereas in Es Trenc, genomospecies represented approximately 90% of the total microbial population (Table S2 and S4).

**TABLE 1:** Statistics of genomospecies representative MAGs recovered from the metagenomes from Es Trenc and Aran-Bidgol inoculum brines and samples at the end of the experiment

MAG	Genomospecies^*a*^	Nr.^*b*^	Origin	Compl. (%)	Cont. (%)	Genome size (Mb)	GC (%)	16S RNA gene(s)
	*Es Trenc*							
**C1**	Genus QS-4–70-19 (f. *Haloferacaceae*)	4	CZ	92.3	0	2.94	70.6	X
**C2**	Genus *Halorubrum* sp.	1	CZ	61.5	0	0.96	69.8	
**C4**	Genus *Halobaculum* sp.	1	CZ	88.5	3.8	1.91	67.5	
**C8**	Genus *Halovenus* sp.	5	CZ	100	0	2.12	65.8	X
**C9**	Species J07HR59 (f. *Haloferacaceae*)	8	CZ	100	0	3.57	58.9	
**C11**	Genus *Halorubrum* sp.	2	CZ	73.1	3.8	2.29	68.4	
**C12**	Genus *Halobellus* sp.	2	CZ	100	0	2.43	67.3	
**Cf2**	Genus A07HB70 (f. *Haloferacaceae*)	7	CCV1	92.3	0	2.54	70.7	
**Cf12**	Genus *Nanosalina*	3	CCV2	80.8	0	0.9	44.2	
**Cf17**	Genus *Halohasta* sp.	1	CCN1	53.8	3.8	3.02	60.9	X
**Cf25**	Genus *Haloquadratum* sp.	9	CCN2	100	3.8	3.84	49.6	
**Cf39**	Species *Haloquadratum walsbyi*	4	CAN1	96.2	0	2.12	47.4	
**Cf40**	Species *Haloquadratum walsbyi*	5	CAN1	100	0	3.65	48.1	
**Cf42**	Genus *Nanosalina*	1	CAN1	50	3.8	0.7	40.0	
**Cf43**	Genus SG9 (f. *Nanosalinaceae*)	1	CAN1	50	0	0.44	46.7	
**Cf44**	Genus *Nanosalinarum*	4	CAN1	80.8	3.8	0.95	56.0	X
**Cf46**	Species *Salinibacter ruber*	7	CAN1	98.1	3.8	3.16	67.2	
**Cf47**	Genus *Halonotius* sp.	1	CAN1	50	0	1.71	61.6	
	*Aran-Bidgol*							
**A1**	Species *Salinibacter ruber*	2	AZ	95.3	3.8	2.85	67.4	
**A2**	Genus *Salinibacter* sp.	2	AZ	73.6	2.8	3.45	65.6	
**A3**	Family *Bradymonadaceae*	1	AZ	79.2	1.9	2.65	62.8	
**A4**	Genus *Halovenus* sp.	1	AZ	73.1	3.8	2	63.5	
**A5**	Family *Haloferacaceae*	1	AZ	46.2	0	1.41	65.9	
**A7**	Genus *Halomicroarcula* sp.	1	AZ	80.8	3.8	1.23	63.3	
**A8**	Genus *Halovenus* sp.	1	AZ	53.8	0	0.83	59.1	
**Af1**	Species *Haloquadratum walsbyi*	3	ACV2	100	0	5.61	49.8	
**Af2**	Family *Bradymonadaceae*	1	ACN1	61.5	0	3.2	61.3	X
**Af8**	Genus *Halonotius* sp.	1	AAV1	73.1	0	1.9	61.9	
**Af10**	Genus *Halovenus* sp.	1	AAV2	88.5	0	2.17	66.6	
**Af11**	Genus *Halonotius* sp.	2	AAN1	92.3	0	1.86	64.8	
**Af13**	Genus *Natronomonas* sp.	4	AAN2	96.2	3.8	2.61	64.2	
**Af14**	Genus *Natronomonas* sp.	2	AAN2	100	0	3.06	63.4	

^*a*^ Lowest taxonomic rank classification.

^*b*^ Number of MAGs conforming the genomospecies.

**Fig 2:**
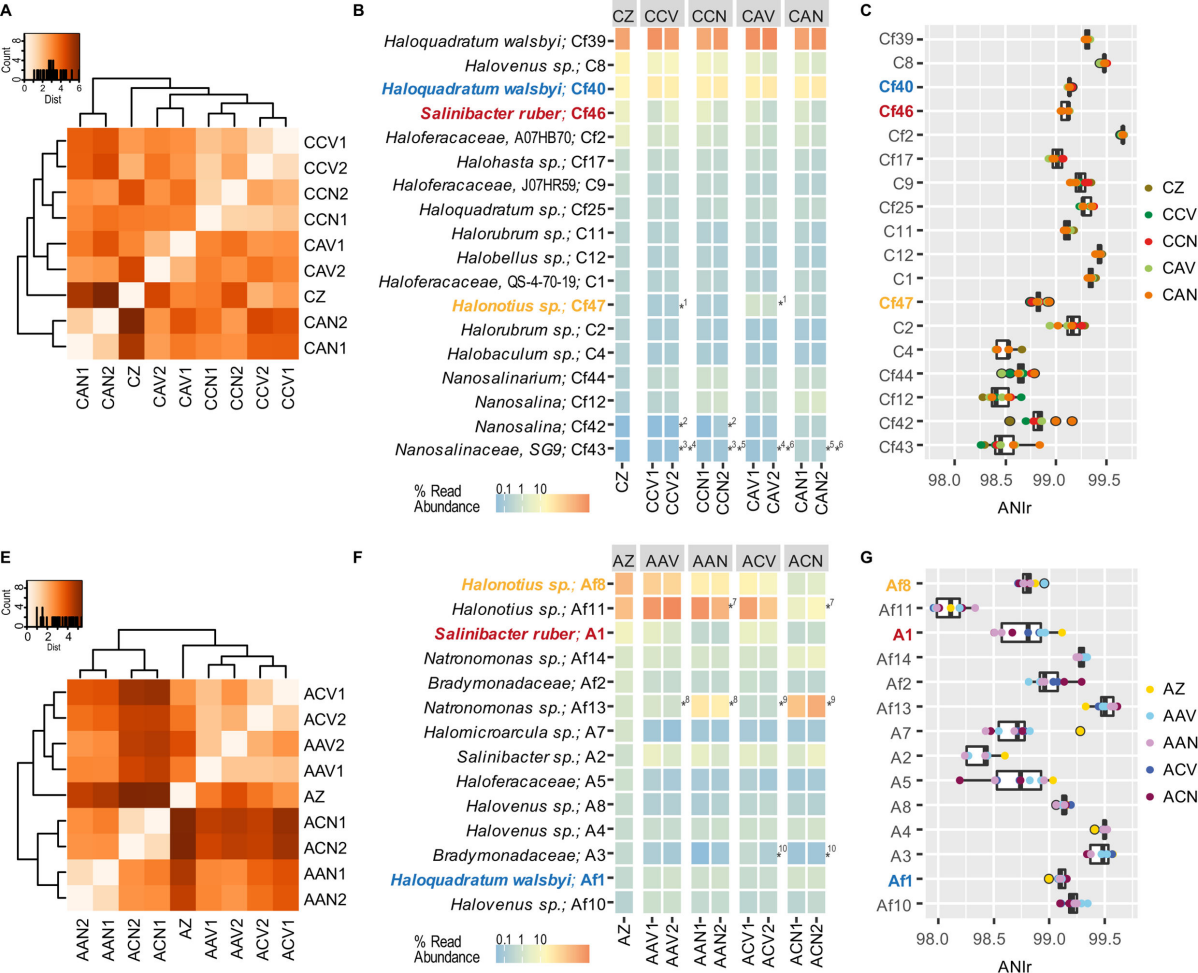
(**A and E**) Heatmap with hierarchical clustering of samples using the variance-stabilizing transformed distance of count data for the representative MAGs. (**B and F**) Heatmap representing MAGs relative abundances in metagenomes in Es Trenc and Aran-Bidgol, respectively. (**C and G**) ANIr at 95% identity and 90% coverage of MAGs in Es Trenc and Aran-Bidgol metagenomes, respectively. MAGs abundance is expressed as percentage of metagenome reads recruited to MAGs and normalized by the total of reads in each metagenome and the total MAG genome size (Mb). Asterisks in (B) and (F) indicate statistically significant differential abundances between comparisons (log_2_ fold change > |1.8|, *P* < 0.05).

After 1 month of incubation, the experimental manipulation impacted Es Trenc and Aran Bidgol MAG community fractions differently. MAGs retrieved from the microcosms inoculated with Es Trenc cells overall did not display important changes in relative abundance when compared to the inoculum, except for endpoints with Aran-Bidgol brines without viruses (CAN, Fig. 2A) and MAGs displaying low abundances (Fig. 2B). Replicate endpoints clustered together (Fig. 2A) with low dissimilarities between samples at the end of the experiment (0.05–0.24 Bray-Curtis index) and between replicates (<0.1, Table S6), which agreed with MASH distance results (dissimilarity of the entire community). Dominant MAGs Cf39 and Cf40, affiliated to *Hqr. walsbyi*, did not display changes in normalized relative abundance due to the environmental exchange, with an average of 11% and 4%, respectively, at the end of the experiment (Fig. 2B; Table S4). Some genomospecies, such as *Halovenus* sp. (MAG C8), decreased in relative abundance at the end of the experiment, especially when incubated in their alien Aran-Bidgol brines where the abundance dropped by half, independently on whether viruses were included or not (Table S4); however, no statistically significant differences were observed. Other MAGs significantly increased as a result of the environmental exchange, like *Halonotius* sp. (MAG Cf47) or *Nanosalinaceae* (MAG Cf43) in the alien Aran-Bidgol brines (log_2_ fold < |1.8|, *P* values < 0.05, Table S4). Overall, low abundant members of the *Nanohaloarchaea* class (MAG Cf42, Cf43) displayed statistically significant abundance increases in any kind of brines without viruses (Fig. 2B).

In contrast, microcosms inoculated with Aran-Bidgol cells exhibited notable changes in MAG community structure due to the environment exchange (Fig. 2E and F; Table S4); these changes were reproducible as endpoint samples clustered together (Fig. 2E). Compared to the samples inoculated with Es Trenc cells and considering MAG composition, Aran-Bidgol samples showed higher overall dissimilarity among them, both along the experiment (0.33 Bray-Curtis index) and within replicates (0.14). Additionally, dissimilarity increased particularly when the relative abundance of MAGs in endpoints with and without viruses was compared (>0.5, Table S6), also observed in the clustering pattern of samples (Fig. 2E), which contrast the lack of a pattern in the dissimilarity between Aran-Bidgol samples when the entire community was considered with MASH distance calculation. The two most abundant genomospecies in Aran-Bidgol, Af8 and Af11, affiliated with the *Halonotius* genus, presented opposite abundance changes at the end of the experiment, decreasing Af8, especially in the alien Es Trenc brines, while increasing Af11, except for the alien Es Trenc brines without virus (ACN). In the case of Af11, this MAG displayed a statistically significant higher abundance in the native brine (log_2_ fold < |1.8|, *P* values < 0.05, Table S4). The genomospecies *Halonotius* sp. (Af13) displayed a significant abundance increase in brines without viruses, whereas the opposite was observed for *Bradymonadaceae* (A3) with significantly lower abundances in alien brines without viruses (Fig. 2F). Other genomospecies displaying notable abundance changes at the end of the experiment were *Sal. ruber* (MAG A1), decreasing especially in brines without virus, or *Salinibacter* sp. (MAG A2) with an overall increase.

The larger community structure changes undergone in Aran-Bidgol cell assemblages compared to that of Es Trenc were not only observed at coarse taxonomic level but also at finer scales of diversity. This was evidenced by the different values of the ANIr recruited to MAGs from either site (Fig. 2C and G), with some MAGs displaying a higher dispersion of ANIr (i.e., A1, A5, and A7) or others showing ANIr in AZ in one of the value extreme ranges, either increasing or decreasing during the experiment.

### Cellular assemblages from Aran-Bidgol and Es Trenc experienced different viral predation pressures

Because viral infection is an important factor structuring prokaryotic communities, both extracellular and intracellular viral genomes were predicted and analyzed to assess not only the extracellular virome but also those viruses potentially infecting cells (i.e., replicating intracellularly), which were present as part of the inocula when the experiment was set up (viruses infecting inoculum cells) and during the incubation in the microcosms. A total of 196 non-redundant bona fide viral genomes were obtained from the viral metagenome from Aran-Bidgol (Table S7), with 188 classified as “quite sure” viral genomes and 57 as “very sure” viral genomes. In the case of the cellular metagenomes (Table S8), a total of 73 non-redundant bona fide viral genomes were retrieved, with 57 classified as “quite sure” and 23 as “very sure” viral genomes (see Methods for selection criteria). It was not possible to recover the metavirome from the inoculum sample from Es Trenc due to problems during the sequencing process. For this reason, we assessed the presence in the cellular metagenomes of viruses predicted in a metavirome belonging to a previous sampling performed in 2014 in Es Trenc, but we could not detect any viral genome from 2014 in the Es Trenc sample used for inoculum here.

**Fig 3:**
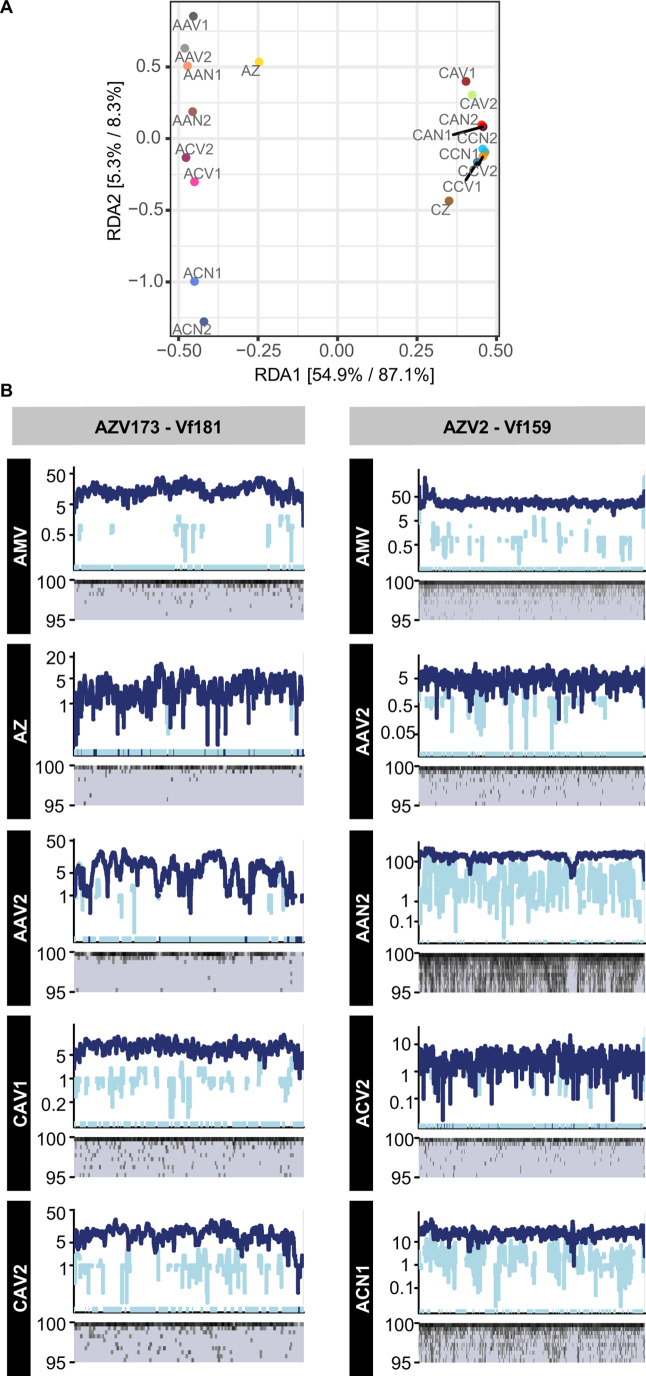
(**A**) db-RDA ordination of abundances of viruses assembled from cellular metagenome using Bray-Curtis dissimilarity and Hellinger transformation, and constrained by the experimental condition (origin of cells and brines, presence and absence of viruses in brines). The relative contribution (eigenvalue) of each axis to the total inertia in the data, as well as to the constrained space only, respectively, is indicated in percent at the axis titles. (**B**) Plot of metagenome reads recruited to viral contigs assembled in the metavirome (sequencing depth in upper panel and identity percentage in lower panel).

To assess changes in the infection pressure exerted by viruses on the cell assemblage caused by the experimental manipulation, viral abundance changes were assessed in cellular metagenomes (Table S7 and S8). This assumed that viral contigs recruiting cellular metagenomic reads correspond to viruses actively replicating inside cells, which could compete with the allochthonous viruses for their target cells, although the detection of (a small fraction of) viruses attached to cell surfaces cannot be ruled out. Richness in Es Trenc viral community showed reproducible results between replicates, whereas the opposite was observed in Aran-Bidgol samples, when accounting for both extracellular viruses (Table S7) and viruses actively replicating (Table S8). This dispersion was evidenced also by distance-based redundancy analysis (db-RDA) (Fig. 3A), with the origin of the cells as the only environmental fitted factor displaying statistical significance (r2 = 0.4038, *P* = 0.02). A similar number (9 and 13 viral genomes, respectively) of viruses assembled from metagenomes were recruited in the cellular fraction in Es Trenc and Aran-Bidgol inoculum, although their abundances were considerably different: from 0.0004% to 0.008% in Es Trenc and from 0.05% up to 0.3% in Aran-Bidgol (Table S8). At the end of the experiment, the relative abundance of viruses in microcosms inoculated with Aran-Bidgol cells was lower than that in the inoculum, decreasing from an average of 20‰ to 0.3–3‰, being especially evident for V14, V12, V13, V11, Vf121, Vf271, Vf205, and Vf181. Regarding V11 and Vf181, both were also detected in Aran-Bidgol metavirome (AZV15 and AZV173, respectively), which confirmed that these viruses were infecting the inoculum cells but nearly disappeared in endpoints (Table S7 and S8). The opposite occurred in microcosms inoculated with Es Trenc cells, where the relative abundance of replicating viruses increased at the end of the experiment, from an average of 0.5‰ in the inoculum to 2‰–3‰, especially for V19 (Table S8).

The annotation of the viral contigs retrieved from viral metagenomes (extracellular viruses) and cellular metagenomes (viruses actively replicating) displayed a high number of hits in NCBI-nr database (10% of total ORFs) related to either haloviruses, both of bacteria and archaea, or their halophilic hosts previously recovered from another hypersaline system. In agreement, most viruses were affiliated with haloviruses (Fig. S4). Among the genes with hits in databases (40%) in the viral contigs, around 70% for both viral and cellular metagenomes, respectively, had their best matches with hypothetical proteins. For the rest of the genes, the presence of genes coding for the synthesis of queosine both in the viral and cellular metagenomes was noticeable, closely related to that of HCTV-1, infecting *Haloarcula californiae*. In addition, some viral contigs (AZV59, AZV19, AZV41, and AZV22) harbored genes coding for the ribosomal protein L32, related to that of some haloarchaea albeit with relatively low identity (from 34.6% to 62.2%). The presence of integrases in viruses retrieved from the cellular metagenomes, indicating the presence of putatively temperate phages, was relatively low (below 0.01%) in both systems. Indeed, 74% of the viruses analyzed were classified as virulent and 25% were temperated (similarly, 75% and 22.5% of viruses retrieved from the viral metagenome were classified as virulent and temperated, respectively). Hosts were assigned for a low number of viral genomes, with only 5 and 7 virus–host pairs from the the Aran-Bidgol metavirome and cellular metagenomes, respectively (2.5% and 9.6% of total viruses), showing an agreement of at least two analytical tools (at a genus and/or genomospecies level, Table S7 and S8).

The response of cellular assemblages to the “infection” with allochthonous viruses was different for both inocula. Although the abundance of viruses in samples with Aran-Bidgol cells amended with allochthonous viruses decreased in one order of magnitude, viral genomes in Es Trenc samples with allochthonous viruses displayed similar relative abundances between endpoints (Table S8). There were some allochthonous viruses, which infected cells from Es Trenc, such as Vf181, Vf185, Vf204, and Vf205 assembled from cellular metagenomes (Table S8) or AZV75 and AZV297 assembled from Aran-Bidgol metavirome (Table S7). The virus Vf181 was also detected in the Aran-Bidgol metavirome, confirming its presence in the extracellular fraction and thus its ability to infect Es Trenc cells in the CAV samples. This infection was accompanied by an increase of Vf181 microdiversity in the corresponding metagenomes, as evidenced by the lower identity percentage of the reads that mapped to the viral contig (Fig. 3B) and by the decrease in ANIr (Table S8). We observed that higher abundance values were obtained in samples from microcosms without extracellular viruses for some viruses, which also showed an increase in microdiversity at the end of the experiment (Table S7 and S8). This was the specific case of the virus Vf159, which was also present in their metavirome of origin. This virus displayed a remarkable increase in microdiversity in metagenomes from endpoint samples with brines lacking the viral suspended fractions, as observed in the recruitment plots (Fig. 3B).

### Autochthonous and allochthonous viruses caused fluctuations in genetic variants in some MAGs

Changes in the abundance of genes in MAGs along the different metagenomes were used to assess fluctuations of variants within a given MAG due to the environmental exchange. The variability in abundance of genes was lower in Es Trenc than in Aran-Bidgol, as observed with db-RDA ordination based on the Bray-Curtis dissimilarity of the sequencing depth of metagenomics reads recruited to MAGs contigs (Fig. S5 and S6). Overall, the presence or absence of viruses in brines caused larger differences in the MAGs gene content in Aran-Bidgol, and these changes were generally reproducible between endpoints in the same conditions as shown by the similarity in the recruitment plots of replicates (Fig. S7).

Aiming to identify genes in MAGs with statistical differences due to the environmental exchange, the abundance fold change of each gene in each metagenome was assessed for selected MAGs, with special interest in genes related with mobile genetic elements, viral infection protection mechanisms, stress, or transport. Contrary to MAGs in Es Trenc, MAGs from Aran-Bidgol displayed significant single-gene fold changes (log_2_ fold > |2|, *P* < 0.05) mainly due to the presence/absence of autochthonous and/or allochthonous viruses in numerous transposases, integrases, ABC transporters, CRISPRs spacers, or genes related with the cell surface (Table 2; Table S9).

**TABLE 2:** MAG gene abundance differences between endpoints assembled in the presence/absence of autochthonous/allochthonous viruses in brines^*a*^

	CCV vs CCN		CAV vs CAN	
MAG, closest relative	↑CCV (%)	↑CCN (%)	↑CAV (%)	↑CAN (%)
Cf40, *Haloquadratum walsbyi*	0.00	0.13	0.00	0.13
Cf46, *Salinibacter ruber*	0.00	0.00	0.00	0.00
Cf47, *Halonotius* sp.	0.00	0.00	0.00	0.65
Cf39, *Haloquadratum walsbyi*	0.00	0.00	0.00	0.24
Cf25, *Haloquadratum* sp.	0.05	0.72	0.02	1.57
Cf12, *Nanosalina* sp.	0.09	0.00	1.20	0.00
C9, J07HR59 (f. *Haloferacaceae*)	0.00	0.11	0.00	0.03

	AAV vs AAN		ACV vs ACN	
	↑AAV (%)	↑AAN (%)	↑ACV (%)	↑ACN (%)
Af1, *Haloquadratum walsbyi*	0.00	0.74	6.36; *CRISPR (2), integrase (6),* *O-antigen (1), transposase (6),* *ABC transporter (1*)	1.46; *CRISPR (2), transposase (2*)
A1, *Salinibacter ruber*	0.00	0.00	0.00	0.00
Af8, *Halonotius* sp.	0.00	0.05	0.00	0.18
A2, *Salinibacter* sp.	0.29; *surface Ag (1*)	0.06	1.83; *transposase (3), ABC transporter (1*)	0.06
Af11, *Halonotius* sp.	0.00	0.34; *transposase (3*)	0.00	0.43; *transposase (3*)
Af13, *Natronomonas* sp.	1.03	0.00	3.35; *transposase (1*)	0.00
Af14, *Natronomonas* sp.	0.00	5.35; *transposase (5*)	0.53; *transposase (1), ABC-transporter (3*)	4.10; *integrase (1), transposase (6*)

^*a*^ Genes with log_2_ fold change >2 and *P* < 0.05 are displayed as percentage of total genes. The number of genes related with infection significantly higher in a given condition are shown.

**Fig 4:**
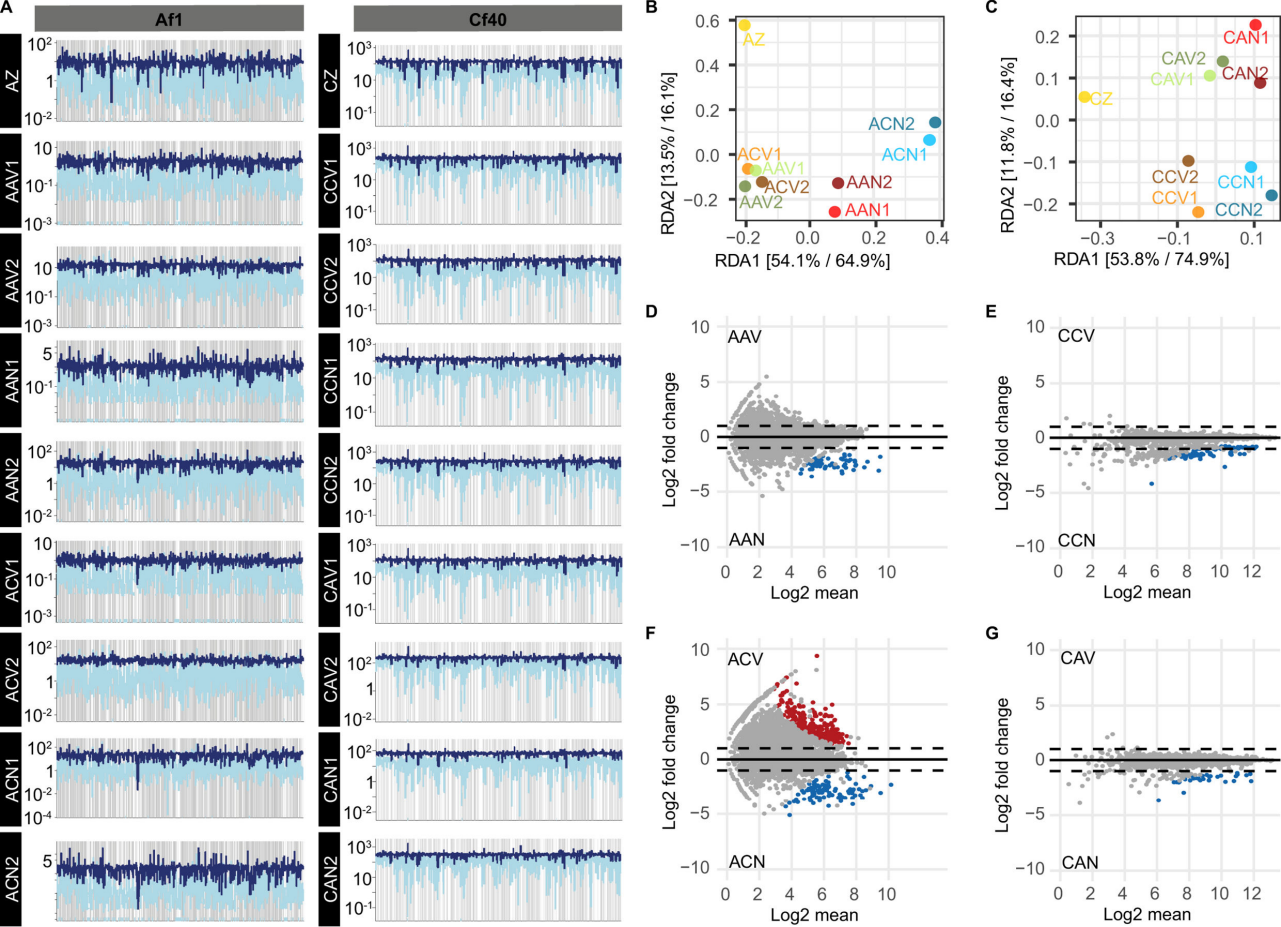
(**A**) Plot of the sequencing depth of metagenome reads recruited to MAGs genomes of representative of the genomospecies *Hqr. walsbyi* in Aran-Bidgol (Af1) and Es Trenc (Cf40). (**B and C**) db-RDA ordination of Es Trenc MAGs based on the Bray-Curtis distance of the sequencing depth of MAG contig genome windows (*n* = 1.000) in each metagenome, normalized by the MAG’s sequencing depth. The experimental conditions (origin of the cells and brines, and the presence/absence of viruses) were used as constraining variables. The relative contribution (eigenvalue) of each axis to the total inertia in the data as well as to the constrained space only, respectively, is indicated in percent at the axis titles. (**D–G**) MA plot showing the log_2_ fold changes of gene counts over the log_2_ mean comparing endpoints with the presence/absence of autochthonous (**D and E**) and allochthonous (**F and G**) viruses for MAGs Af1 (**D and F**) and Cf40 (**E and G**). Genes with statistically significant (*P* < 0.05) log_2_ fold changes > 2 and log_2_ fold changes < −2 are highlighted.

Interestingly, MAGs recovered from Es Trenc and Aran-Bidgol belonging to the same genomospecies (reciprocal ANI > 95%) responded differently to the change of environment and viral infection pressure. This was especially evident for *Hqr. walsbyi* (MAGs Af1 in Aran-Bidgol and Cf40 in Es Trenc). MAG Af1 displayed higher changes in gene content toward the end of the experiment (Fig. 4) independently on the origin of brines, with 6% of genes significantly changing in abundance when growing with allochthonous viruses in Es Trenc brines, whereas MAG Cf40 displayed less than 1% of genes significantly changing in abundance in any of the cases (Table 2). Pangenome analysis of MAGs conforming this genomospecies showed differences in terms of specific genes (Fig. S8), revealing Cf40 to carry genes belonging to the BREX system, which is related to phage resistance. In the case of the shared genomospecies *Sal. ruber* (MAGs A1 and Cf46), although a higher gene content change was observed in Aran-Bidgol (Fig. S9A through G), no statistically significant changes were observed in any comparison (Table 2). Contrarily, the genomospecies *Halonotius* sp. (MAGs Af8 and Cf47) experienced higher changes in Es Trenc than in Aran-Bidgol (Fig. S9H through N), yet these changes occurred in less than 1% of genes (Table 2).

### The lake microbial community displayed greater intraspecific diversity changes than that of the solar saltern

**Fig 5:**
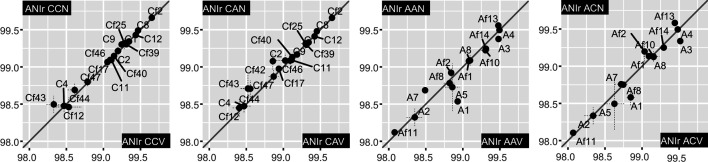
Pairwise comparisons between ANIr of MAGs at endpoint metagenomes, averaged between replicates (range error bars are represented with dashed lines). Only MAGs with a sequencing breath above 70 were considered (70% of genome bases covered by sequencing reads).

Changes in ANIr of MAGs between inoculum and the endpoint metagenomes, and between metagenomes assembled with and without viruses were assessed (Fig. 5; Table S10), aiming to elucidate whether these changes resulted from genetic variant selection or due to an increase in diversity at an intraspecific level. Overall, ANIr of MAGs from Es Trenc was stable for most (Fig. 2C and 5), when comparing both assemblies with and without viruses in brines and endpoints with the inoculum (median of 0.04 in ANIr absolute differences, Table S10), except in MAGs Cf12 affiliated to *Nanosalina* sp., displaying an overall increase in ANIr at the end of the experiment, especially in Es Trenc brines and their viral fraction. Contrarily, MAGs from microcosms assembled with Aran-Bidgol cells displayed more marked ANIr differences (Fig. 5), with averaged absolute difference of 0.2% (median of 0.1%, Table S10). Microdiversity overall increased for MAGs A7 (*Halomicroarcula* sp.) and A5 (family *Haloferaceae*) at the end of the experiment and specifically for MAG A1 (*Sal. ruber*) in brines without viruses (Table S10; Fig. 5). In contrast, microdiversity decreased in MAGs Af2 (family *Bradymonadaceae*) and Af13 (*Natronomonas* sp.), especially in brines without viruses.

## DISCUSSION

Here, two distant hyperhalophilic microbiomes, from Aran-Bidgol salt lake (Iran) and Es Trenc solar salterns (Campos, Mallorca, Spain), were challenged by transplanting their cellular, brine, and virus fractions, and the effect of the environmental change on the community was studied by means of comparative metagenomics. The results showed that the transplant did not affect the microbiomes in terms of alpha-diversity at the short term (Nonpareil diversity), which was maintained after 1 month. However, even when both microbiomes were dominated by the same taxa, mainly belonging to the *Halobacteria* archaeal class, the more diverse Aran-Bidgol community was highly impacted by the incubation conditions in terms of beta-diversity between endpoint conditions (MASH distance), whereas the less diverse Es Trenc community showed very minor impacts. The dissimilarity displayed by Aran-Bidgol microcosms did not show a clear pattern on whether either the change in brine ionic composition or the viral predation pressure was the main factor affecting community structure when the entire community was considered. However, when only MAGs were considered, beta-diversity mainly increased due to changes in the viral predation pressure. Aran-Bidgol MAG fraction displayed a more complex structure where the impact of the environmental exchange was more noticeable as a result of selective processes influencing an already stressed community. Es Trenc MAG fraction was consistently dominated by *Hqr. walsbyi* and other members of the genus *Haloquadratum*, with *Sal. ruber* as the only bacterial representative, which is in agreement with what we have previously observed in this solar salterns (11, 25). The higher stability displayed by Es Trenc microcosms could be related to the human-controlled cycles of evaporation and refilling occurring in solar salterns, giving rise to more robust microbiomes that are more resistant and resilient to environmental changes, such as fluctuations in salinity and sunlight irradiation (11, 25). Particularly, the microbial community in Es Trenc solar saltern is well adapted to fluctuations and stressful events, as indicated by the stability shown by the community structure, reported by our group over the last 20 years (11, 25–27).

A question that arises is why the solar saltern community members better resisted the environmental exchange, when considering both the entire community and the assembled dominating taxa. It has been often reported that high diversity levels ensure a higher community stability (28–30). However, here, the results indicate otherwise because Aran-Bidgol community displayed a higher alpha-diversity than that of Es Trenc while also displaying higher dissimilarities as indicated by the MASH distance. Additionally, when we considered fine taxonomic ranks in the MAG fraction of the community, we observed a higher response to the experimental conditions in Aran-Bidgol genomospecies, specially caused by changes in the infection pressure in some (i.e., *Sal. ruber*, *Bradymonadaceae* or *Natronomonas* sp.). Interestingly, microdiversity decreased for some genomospecies like *Halomicroarcula* sp. and *Haloferaceae*, which might be related to the reduction or disappearance of selection forces due to the incubation conditions. Contrarily, the Es Trenc community was more resistant toward the experimental manipulations, which suggests that the solar saltern contained a well-adapted community. It has been reported that the existence of different ecotypes provides community stability and ensures the persistence of higher taxonomic levels in different ecosystems such as freshwater lakes, seawater, or soils (4, 5, 10). We have also previously observed the coexistence of distinct ecotypes adapted to different salt concentrations in the dominant *Hqr. walsby* in Es Trenc solar saltern, which persisted under changing salinities (11). Here, the intraspecific genotypic diversity previously observed in Es Trenc microbial community might have buffered the effects of the environmental exchange as we had shown previously for *Sal. ruber* (12). The reasons behind the maintenance of this genotypic diversity remain, however, elusive. A possible explanation could be the combination of higher recombination rates within ecotypes and a relative weak selection that would avoid genome-wide selective sweeps (31). The maintenance of such genotypic diversity could then be considered as an evolutionary strategy to ensure the long-term ecological success of genomospecies inhabiting highly fluctuating conditions (14), such as solar salterns. Additionally, top-down diversity control exerted by viruses such as KtW interactions could be behind the high microdiversity observed in hypersaline habitats, which could be even sufficient to prevent diversity purges.

In agreement with the large changes observed in Aran-Bidgol prokaryotic community structure, viruses also displayed considerable abundance changes within microcosms. Viruses recruited in the inoculum cellular metagenomes, and thus infecting cells, of Aran-Bidgol showed relatively higher abundances than in Es Trenc even when brines from both hypersaline ecosystems displayed similar VLP. The viral community in Aran-Bidgol metavirome evidenced the presence of genes related to viral propagation strategies, such as genes encoding a ribosomal protein L32, thought to reprograming the host cell metabolism (32), or genes coding for the synthesis of queosine, reported in the genomes of diverse viruses (including the halovirus HVTV-1) as a means of genome modification for evasion of the host restriction-modification system (33).

It seems that the viral community in Es Trenc was highly specialized as Aran-Bidgol cells were resistant to infection by these allochthonous viruses. The highly specialized prokaryotic community found in solar salterns would explain the high specificity shown by the accompanying viral community (34, 35). It was observed for some viruses (i.e., AZV173-Vf181, AZV2-Vf159) that microdiversity increased when the infection pressure was artificially reduced by the experimental manipulation. The high virus–host specificity observed in Es Trenc did not occur with Aran-Bidgol virome as some of its viruses were recruited in Es Trenc cellular metagenomes, indicating that there were sensitive hosts toward Aran-Bidgol viral infection. The ability of allochthonous viruses to infect cells supports the idea of a global exchange of hosts and viruses in hypersaline environments (34).

We sought to investigate the influence of viruses in structuring gene content and turnover among subpopulations. We have previously shown that studies on population genome binning can be used to assess shifts in gene content in sequence-discrete populations and their ecological implications in abundant members of the community (11, 12, 25). The quantification of gene content diversity in sequence-discrete populations can then be used as a proxy of fluctuations of genetic variants comprised within MAGs (12, 36). Here, we observed the dispersion in gene turnover increased in brines lacking the viral suspended fraction, which is consistent with the importance of viral predation selective pressure on gene turnover in MAGs. In such circumstances, the host top-down regulation exerted by viruses was no longer present for some MAGs, as postulated in the KtW hypothesis (18). It should be taken into consideration that an unknown proportion of phages adsorbed to their hosts could have partially contributed to the observed effects in our experiments. Nevertheless, the applied change in viral predation pressure was enough to uncover an increase in abundance in mobile genetic elements and genes involved in cell immunity and cell surface in Aran-Bidgol-analyzed MAGs. Viral predation exerts a negative frequency-dependent selection in genes encoding for cellular immunity and cell surface structures targeted by phages (18). These genes are often localized in genomic islands, which typically encode transposases and integrases involved in genetic turnover by means of non-homologous recombination (14). This was especially evident in the shared genomospecies between both microbiomes *Hqr. walsbyi* (MAGs Cf40, Af1), which displayed a genetic turnover for these genes in MAG Af1, whereas no genetic turnover was observed for Cf40. Analysis of the pangenome revealed Cf40 to carry genes belonging to the BREX system, which has been reported to confer resistance to a broad range of phages by blocking viral replication (37–39) and could have contributed to the stability shown by this MAG toward changes in the viral infection pressure.

Here, we showed that a solar saltern community adapted to recurrent environmental changes was able to withstand changes both in environmental physicochemical composition and in viral predation pressure. By manipulating the viral predation pressure by autochthonous and allochthonous viruses, we show their important role in shaping the microbial community structure at a genetic variant level in some MAGs. Bearing in mind that MAGs are population consensus genomes (21), we studied the gene content fluctuations within MAG populations, which evidence changes in the dominance of different strains (36). We showed a genetic turnover and microdiversity variability within sequence-discrete populations mainly caused by viruses, especially in the lake community, which was less adapted to highly fluctuating conditions.

## MATERIALS AND METHODS

### Experimental design

Cellular fractions were obtained by centrifugation (20,000 rpm). The supernatants were filtered first with 0.22-µm Sterivex filters and then with the tangential-filtering using Vivaflow 200-PES system obtaining two fractions, one filtered with 500 mL of the brine without the free viruses originally present in the sample, and the remaining 500 mL twofold enriched in viruses. Each of the cellular Es Trenc and Aran-Bidgol fractions was resuspended in brines (with or without free viruses, and same or different location) and was split into 75-mL duplicate microcosms and incubated for 33 days (Fig. 1A). The ionic composition of the original brines and endpoint brines at the end of the experiment was assessed by ion chromatography, and salinity of the inoculum samples was measured with a hand refractometer. Bacteria and archaea were assessed by CARD-FISH microscopy as described by Viver et al. (40). Viral quantification of inoculum brines was performed as described by Boujelben et al. (41). Viral morphologies were determined by transmission electron microscopy. For a detailed description, see Supplementary Methods and Figure S1.

### Metagenome and metavirome sequence processing

Metagenome DNA extraction was performed from 34-mL volume brine samples using the phenol chloroform isoamyl alcohol (PCIA) method as detailed by Urdiain et al. (42). Samples for metavirome analysis were preprocessed as described by Font-Verdera et al. (43), obtaining viral pellets by ultracentrifugation. Viral particles were embedded in agarose plugs, and DNA was extracted as specified by Santos et al. (44). Full details are given in the Supplementary Methods. DNA samples were sequenced using Illumina HiSeq (Table S2). Cellular contamination in the metavirome sequences was assessed by searching both archaeal and bacterial 16S rRNAs with Parallel-META v3.4 (45) and default settings, validating the metavirome when less than 0.02% of the reads belonged to 16S rRNA genes (46). For both metagenome and metavirome, paired-end reads were trimmed with BBduk v38.82 (47) with ktrim = r mode, k = 28, mink = 12, discarding reads with a quality score below 20 and a length below 50 or 100 bp (for 2 × 100 and 2 × 150 bp runs, respectively, Table S1). The metagenome/metavirome coverage and sequence diversity was assessed by Nonpareil analysis (48), and MASH distance was computed using k = 32 and a sketch size of 1,000,000 (49). A PCoA of the MASH distance was performed using Ape R package (50). Trimmed reads were joined using the Enveomics collection script *FastA.interpose.pl* (51) and assembled with the IDBA v1.1.3 assembler (52) using the pre-correction mode. Genes from contigs with lengths of ≥500 and 10,000 bp for metagenomes and metaviromes, respectively, were predicted using Prodigal v2.3.6 (53).

### MAG recovery and analysis

Contigs with a length of ≥2,000 bp were binned using MaxBin v2.2.7 (54). Bins were manually refined with Anvi'o v6.2 (55). Completeness and contamination of resulting MAGs were calculated using the Microbial Genomes Atlas tool, MiGA (56). Phylogenetic analysis of MAGs was performed using the GTDB-tk v2.1.1 (Release 207_v2) tool with the *classify_wf* pipeline (57). Sequence metrics and ANI between genomes were obtained with scripts from the Enveomics collection (51). The abundance of MAGs in each metagenome was calculated by the recruitment with BLASTn (58) of metagenomics reads filtering by *BlastTab.best_hit_sorted.pl* (51) and with ≥95% similarity and alignment length of ≥90% to the genome and represented with heatmaps performed with ampvis2 R package (59). The relative abundance of each species was calculated, normalizing the number of reads mapping to the MAG genomes by both the total number of reads in the metagenome and the genome length. Genome equivalents were obtained with the MicrobeCensus software (60), and genomospecies abundances were obtained by normalizing representative MAGs sequencing depth by the genome equivalents. Count data for MAGs were normalized using variance-stabilizing transformations with DESeq2 (61), and a hierarchical clustering was performed with heatmap.2 function in the gplots package (62). A differential abundance analysis of MAGs counts between the different experimental conditions was performed with DESeq2 (61) using Wald tests (Benjamini–Hochberg [BH] adjusted *P* value < 0.05). Recruitment plots, sequencing depth and breadth, and ANIr were calculated with enveomics.R R package (51), with ANIr being the average nucleotide identity of reads against a genome and the sequencing breadth being the percentage of genome bases sequenced at a given sequencing depth. Contig recruitment was used to identify subpopulations (*enve.recplot2.findPeaks*), and the sequencing depth of all MAG contig genome windows (*n* = 1.000) in each metagenome was extracted (enve.recplot2.extractWindows), normalized by the MAG average sequencing depth in that metagenome, and a Bray-Curtis distance-based RDA constrained by the experimental condition was performed with ampvis2 R package. Python scripts of pipelines described by Roth E. Conrad, available at https://github.com/rotheconrad, were used to annotate protein-coding genes with BLASTp (58) against TrEMBL and Swissprot databases (63), filtering by ≥40% identity, ≥50% coverage, and ≤0.01 e-value, and for pangenome analysis of selected MAGs. Abundance of MAG genes in each metagenome was calculated by competitive best-match recruitment of metagenomic reads to the genome (≥95% similarity and ≥90% coverage) using BLASTn (58). Changes in MAG gene abundance in each metagenome were identified with DESeq2 (61) using count data for all genes in the genome and independently run on each individual MAG using Wald tests (BH adjusted *P* value < 0.05), and MA plots were generated with ggpubr (64).

### Identification of bona fide viral contigs and analysis

Considering the prevalence of archaeal viruses in hypersaline environments, a relaxed viral contig prediction followed by a manual curation was followed, aiming to maximize novel diversity capture. Viral contigs with a length of ≥10,000 bp were predicted from the metavirome and metagenomes as either viral or prophage genomes by VirSorter (65), and were clustered with Cd-hit v4.7 into non-redundant viral contigs with cdhit-est -c 0.9 –n 8 (66). A manual curation of viral contigs was performed to be further considered as bona fide viruses, based on criteria previously described by Ramos-Barbero et al. (67). Briefly, viral contigs should be included into one of the VirSorter categories (1–6) and display ≥8% of annotated genes as viral by DIAMOND BLASTp (68) against NCBI-nr database (e-value < 0.0001). The bona fide viral genomes were additionally analyzed with VirSorter2 (69), PhaTYP (70), and PhaBOX (71), and were classified as “quite sure” and “very sure” of being correctly classified as bona fide virus according to the agreement of two or three tools, respectively. Bona fide viral genomes were additionally annotated with InterProScan v5.50-84.0 (72) using Pfam (73), TIGRFAMs (74), CDD (75), and SMART (76) databases. Viral lifestyle was assessed with PhaTYP (70) and the inspection of integrases in genomes. Abundance of viruses in the metavirome and in each metagenome was calculated by competitive best-match recruitment to the genome (≥95% similarity and ≥90% coverage) using BLASTn. The abundance dispersion was evaluated with db-RDA ordination with ampvis2 (ordination functions wrapped around vegan R package), calculating the Bray-Curtis distances between endpoints of Hellinger transformed abundance data and constraining by the origin of the cells and brines and the presence/absence of viruses in reconstituted brines. Recruitment plots sequencing depth and breadth and ANIr were calculated with enveomics.R. The presence of viral contigs in each metagenome was considered when the sequencing breadth was higher than 70% and by further visual inspection of recruitment plots. Viral genomic sequences were uploaded to the ViPTree v3.5 (77) and were visualized using iTOL v6.7.4 (78). Virus–host prediction was explored using a suit of computational tools, which included iPHoP (79), PHIST (80), prokaryotic virus host predictor (81), applying default settings. Additionally, CRISPR spacers were predicted with CRISPRCasFinder (82), and BLASTn comparisons were performed, considering virus–host associations in alignments with ≥90% similarity and ≥90% coverage. Viral and host tRNAs were predicted with tRNAScan (83) using the bacterial/archaeal models, and BLASTn comparisons were performed, considering virus–host associations in alignments with ≥90% similarity and ≥90% coverage. Furthermore, viral contigs were searched in MAGs using BLASTp (≥95% similarity and ≥90% coverage).

## Data Availability

The data sets generated during the current study are available in the European Nucleotide Archive (ENA) repository with project accession number PRJEB54903.

## SUPPLEMENTAL MATERIAL

Supplemental Material msystems.00538-24-s0001.pdf Supplemental methods, tables, and figures.

Table S3 msystems.00538-24-s0002.xlsx MASH distance between metagenomic reads of inoculum brines and microcosms at the end of the experiment in Es Trenc and Aran-Bidgol samples.

Table S4 msystems.00538-24-s0003.xlsx Statistics of MAGs recovered from metagenomes from Es Trenc and Aran-Bidgol brines and microcosms samples.

Table S7 msystems.00538-24-s0004.xlsx Statistics of viral genomes recovered from the metavirome from Aran-Bidgol inoculum.

Table S8 msystems.00538-24-s0005.xlsx Statistics of viral genomes recovered from metagenomes from Es Trenc and Aran-Bidgol brines and microcosms.

Table S9 msystems.00538-24-s0006.xlsx Log2-fold gene abundance differences of selected MAGs between microcosms.

ASM does not own the copyrights to Supplemental Material that may be linked to, or accessed through, an article. The authors have granted ASM a non-exclusive, world-wide license to publish the Supplemental Material files. Please contact the corresponding author directly for reuse.
