# Supplementary material for: Extremely halophilic brine community manipulation shows higher robustness of microbiomes inhabiting human-driven solar saltern than naturally driven lake
Source: mSystems. 2024 Jun 27;9(7):e00538-24. doi: 10.1128/msystems.00538-24 (PMC11324034; doi:10.1128/msystems.00538-24)
Supplement: Supplemental Material — Supplemental methods, tables, and figures. [file msystems.00538-24-s0001.pdf]

Extremely halophilic brine community manipulation shows higher robustness of microbiomes inhabiting human-driven solar saltern than naturally driven lake

Raquel Liébana[1* #]; Tomeu Viver[1,2]; María Dolores Ramos-Barbero[3,4]; Esteban Bustos-Caparros[1]; Mercedes Urdiain[1]; Cristina López[3]; Mohammad Ali Amoozegar[5]; Josefa Antón[3]; Ramon Rossello-Mora[1]

[1]Marine Microbiology Group, Department of Animal and Microbial Biodiversity, Mediterranean Institute for Advanced Studies (IMEDEA, UIB-CSIC), Esporles, Spain

[2]Department of Molecular Ecology, Max Planck Institute for Marine Microbiology, Bremen, Germany

[3]Department of Physiology, Genetics and Microbiology, University of Alicante, Alicante, Spain

[4]Department of Genetics, Microbiology and Statistics, University of Barcelona, Barcelona, Spain

[5]Extremophiles Laboratory, Department of Microbiology, School of Biology and Center of Excellence in Phylogeny of Living Organisms, College of Science, University of Tehran, Tehran, Iran

*Current Address: AZTI, Marine Research Division, Basque Research Technology Alliance (BRTA), Sukarrieta, Spain

[#] Address correspondence to Raquel Liébana, rliebana@azti.es.

Supplementary methods

Study sites and experimental design.

This study was carried out using 1L of brines from the Mediterranean solar salterns of Es Trenc, located in the municipality of Campos on the southeast coast of Mallorca Island, Spain (39°20′N; 2°59′E) sampled on May 4th 2016. The second 1L sample was obtained from the thalasohaline lake of Aran-Bidgol, in central Iran (34°18′-34°45′N; 51°33′-52°10′E), on February 11th, 2016. The sample of Aran-Bigdol was stored at room temperature in the laboratory under day-light cycle until the experiment start. On May 5th 2016, 960 and 945 ml of brines from Es Trenc and Aran-Bidgol respectively were used to obtain three fractions: cells, brine with viruses and brine without suspended viruses. The cellular fractions were obtained by centrifugation (3x 20,000 rpm, 10 min, 4 ºC). The supernatants were filtered with 0.22 µm Sterivex filters to remove the remaining cells, and further filtered using Vivaflow 200-PES system (300 ml/min) obtaining two fractions, one filtered with 50% of the brine without the free viruses originally present in the sample, and the remaining 50% enriched in viruses. Each of the cellular Es Trenc and Aran-Bidgol fractions were resuspended using its corresponding processed brine (with or without virus free viruses, and same or different location). Then, these mixes were split into 75 ml duplicate mesocosms with cells from each site resuspended in the original or alien brine, with or without the viral fraction (Figure S1). The mesocosms were incubated for 33 days at 30 ºC, with 14:10 h light:dark cycle. The ionic composition of the original brines and endpoint brines at the end of the experiment was assessed by ion chromatography by Technical Research Services of Alicante University (Spain). Salinity of the inoculum samples was measured with a Sper Scientific Salt Refractometer.

Cell and virus microscopy analysis.

The archaeal and bacterial fractions were separately assessed by CARD-FISH microscopy in brine samples fixed with formaldehyde 4% and further processing samples as described in (1). Briefly, samples were centrifuged (13,000 rpm for 5 min), washed twice in 1x PBS and resuspended in ethanol and 1x PBS (1:1) to further dilute 50 µL in 10 ml of 1x PBS and filter with 0.2 µm GTTP filters. CARD-FISH probes used were EUB338-I, -II and -III for Bacteria, (ARCH915 for Archaea, NON338 as negative control and counterstained with DAPI. Microscopy counts were performed with a Axioskop 2 mot plus epifluorescence microscope.

Viral quantification with Sybr-Gold stain was performed on the inoculum brines previously fixed with formaldehyde 4% and as described in (2). Briefly, fixed samples were filtered with 0.02 µm Anodisc 25 filters, and viral particles were stained with Sybr gold and counted with a Leica DM4000B epifluorescence microscope. Viral morphologies were determined with a Jeol JEM-2010 transmission electron microscope. For this, 1 ml of viral concentrates was filtered (0.22 µm pore size) and concentrated

using ultracentrifugation (2x 186,000 g at 20 ºC for 2 h), and sample volumes of 5 µL were stained with 2% uranyl acetate for 45 min.

Metagenome and metavirome sequence processing.

A volume of 34 mL of brine samples was centrifuged at 13,000 rpm for 5 min and the pellet stored at -20 ºC until use. Metagenome DNA extraction was performed as detailed in (3). Briefly, cell biomass was washed with 1x PBS and resuspended in 1ml saline EDTA. Prior to extraction, cells were treated with 3.3 µL of lysozyme (300 mg ml^{-1}), 10 µL of proteinase K (10 mg ml^{-1}) and 10 µL of mutanolysine (1000 units ml^{-1}) for 15-45 min at 37 ºC. Cellular lysis was performed with 80 µL of 25% SDS (10 min at 65 ºC) and subsequently adding 250 µL of 5M NaCl. The mixture was then washed twice with an equal volume of phenol:chloroform:isoamyl alcohol (25:24:1), recovering the aqueous phase by centrifugation (13,000 rpm for 3 min) and the DNA was precipitated by adding 1/9 vol of sodium acetate 3M (pH 7) and 0.6 vol of isopropanol. The final DNA precipitate was dissolved in sterile MilliQ water and kept at -20 ºC until use.

Samples for metavirome analysis were preprocessed as described in (4) and DNA was extracted as specified in (5). Briefly, supernatants were obtained by centrifugation of 250 mL in two successive steps of 13,500 and 20,000 rpm (15-20 min at RT), further filtered through 0.22 µm filters (Sterivex) and concentrated to approximately 40 ml by ultracentrifugation at 286,000 x g (4 h at 20 ºC) in a Beckman Coulter® optimaTM Max-XP ultracentrifuge with a SW Ti 41 rotor. Viral pellets were mixed with 1.6% agarose (1:1) and dispensed into 100 µl molds. Solidified agarose plugs were washed with TE and incubated with 10 units of RQ1 DNase overnight. Viral capsid disruption was further performed incubating the mixture with ESP (0.5 M EDTA, pH 9.0, 1% N-laurylsarcosine, 1 mg ml−1 proteinase K) at 50°C overnight and washed with TE-Pefabloc (10 mM Tris-HCl, 1 mM EDTA pH 8.0, 3 mM Pefabloc) at 65 ºC for 15 min. Thereafter, the agarose was removed with β-agarase (1.5 h at 42 ºC), then purifying the DNA using Microcon Centrifugal Filter Devices YM-100.

References

1. Viver T, Orellana LH, Hatt JK, Urdiain M, Díaz S, Richter M, Antón J, Avian M, Amann R, Konstantinidis KT, Rosselló-Móra R. 2017. The low diverse gastric microbiome of the jellyfish Cotylorhiza tuberculata is dominated by four novel taxa. Environ Microbiol 19:3039–3058.

2. Boujelben I, Yarza P, Almansa C, Villamor J, Maalej S, Antón J, Santos F. 2012. Virioplankton community structure in Tunisian solar salterns. Appl Environ Microbiol 78:7429–7437.

3. Urdiain M, López-López A, Gonzalo C, Busse HJ, Langer S, Kämpfer P, Rosselló-Móra R. 2008. Reclassification of Rhodobium marinum and Rhodobium pfennigii as Afifella marina gen. nov. comb. nov. and Afifella pfennigii comb. nov., a new genus of photoheterotrophic Alphaproteobacteria and emended descriptions of Rhodobium, Rhodobium orientis and Rh. Syst Appl Microbiol 31:339–351.

4. Font-Verdera F, Liébana R, Aldeguer-Riquelme B, Gangloff V, Santos F, Viver T, Rosselló-Móra R. 2021. Inverted microbial community stratification and spatial–temporal stability in hypersaline anaerobic sediments from the S’Avall solar salterns. Syst Appl Microbiol 44:126231.

5. Santos F, Yarza P, Parro V, Briones C, Anton J. 2010. The metavirome of a hypersaline environment. Environ Microbiol 12:2965–2976.

Supplementary material

Figure S1. Scheme of protocol used for the reciprocal transplant of cells, brines and viral fraction between Es Trenc and Aran-Bidgol samples.

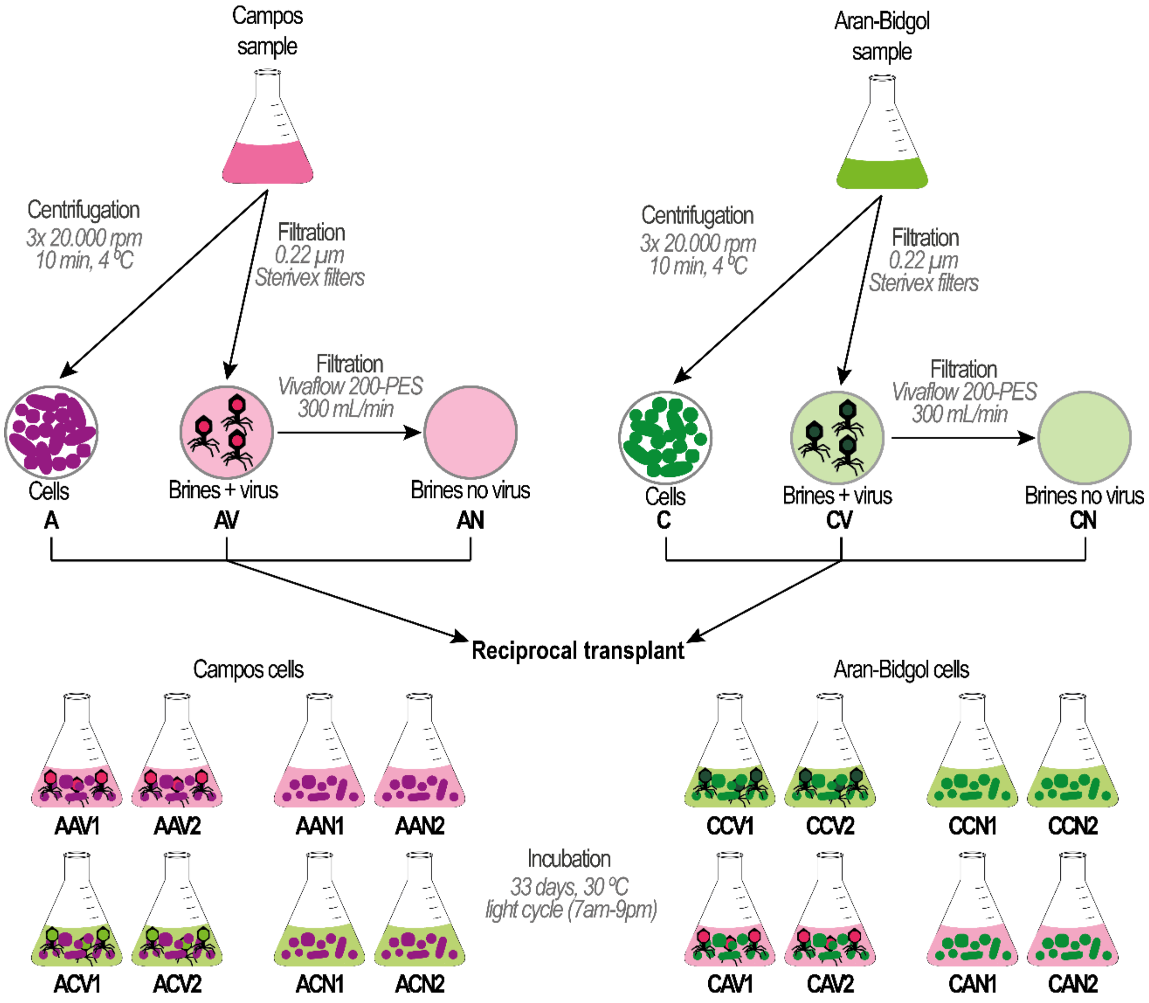

Table S1. Ionic composition of samples from Es Trenc and Aran-Bidgol inoculum brines and from microcosms at the end of the experiment.

Sample	Fl^-	Cl^-	Br^-	NO_3^-	SO_4^{2-}	Li^+	Na^{2+}	K^+	Mg^{2+}	Ca^{2+}
**CZ**	0.011	179.612	0.803	0.001	22.008	0.000	95.598	4.812	15.577	0.482
**CCV1**	0.010	178.603	0.804	0.000	21.592	0.001	95.844	4.881	15.705	0.297
**CCV2**	0.014	312.024	1.409	0.001	38.883	0.001	167.589	8.378	26.679	0.533
**CCN1**	0.013	236.757	1.055	0.001	28.460	0.000	126.822	6.385	20.249	0.440
**CCN2**	0.013	223.861	1.028	0.000	22.684	0.001	116.525	6.174	19.883	0.453
**ACV1**	0.013	215.540	0.966	0.001	26.940	0.002	113.642	5.895	19.436	0.419
**ACV2**	0.013	240.748	1.120	0.001	26.486	0.004	127.080	6.738	21.714	0.446
**ACN1**	0.011	198.551	0.877	0.000	23.801	0.000	107.385	5.363	17.179	0.412
**ACN2**	0.013	284.804	1.323	0.001	34.571	0.002	150.670	7.815	25.232	0.615
**CAV1**	0.005	221.558	0.077	0.000	13.692	0.014	133.363	1.471	9.570	0.920
**CAV2**	0.007	224.340	0.079	0.000	14.216	0.015	134.189	1.494	10.298	0.785
**CAN1**	0.006	189.044	0.070	0.000	11.529	0.013	112.310	1.213	8.294	0.583
**CAN2**	0.006	172.712	0.062	0.001	10.398	0.011	102.539	1.186	7.824	0.486
**AAV1**	0.006	213.156	0.071	0.000	12.999	0.014	127.337	1.349	9.300	0.587
**AAV2**	0.007	208.390	0.066	0.001	12.880	0.011	125.895	1.225	8.417	0.537
**AAN1**	0.006	191.777	0.062	0.000	11.560	0.012	115.589	1.201	8.379	0.570
**AAN2**	0.007	188.645	0.064	0.001	11.504	0.012	112.040	1.211	8.471	0.597
**AZ**	0.006	231.726	0.077	0.000	14.348	0.028	137.279	1.497	10.231	0.642

Figure S2. Selection TEM images from Aran-Bidgol (A) and Es Trenc (B) brines, showing different viral morphotypes.

A

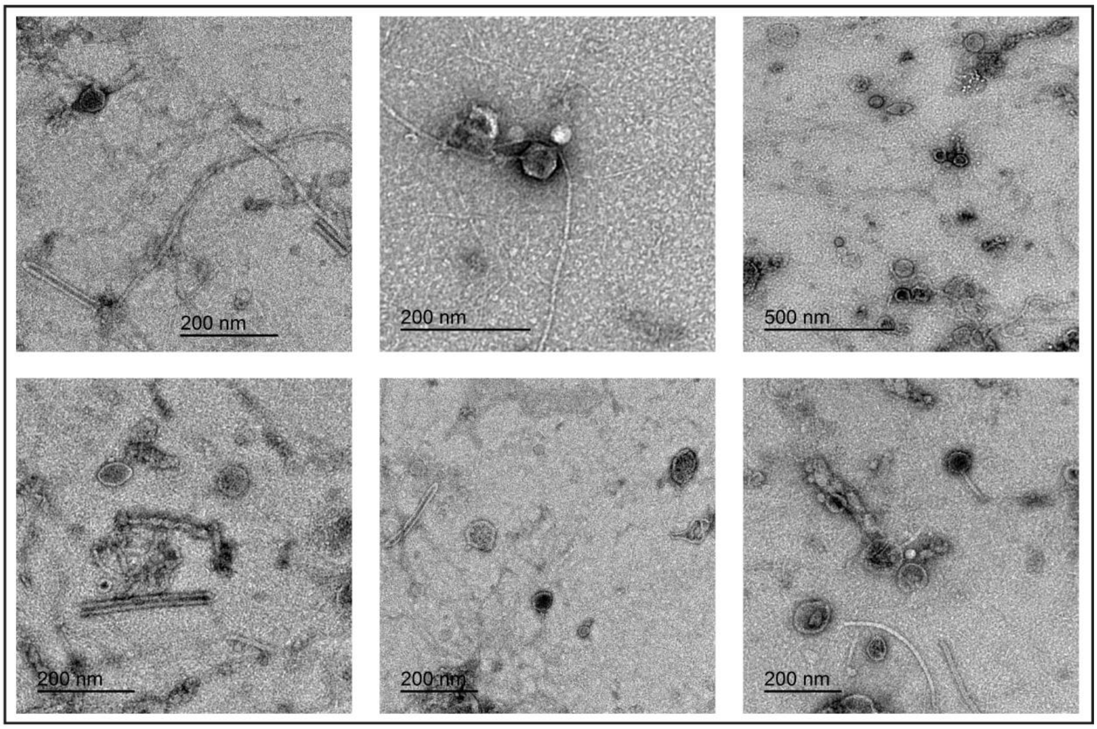

B

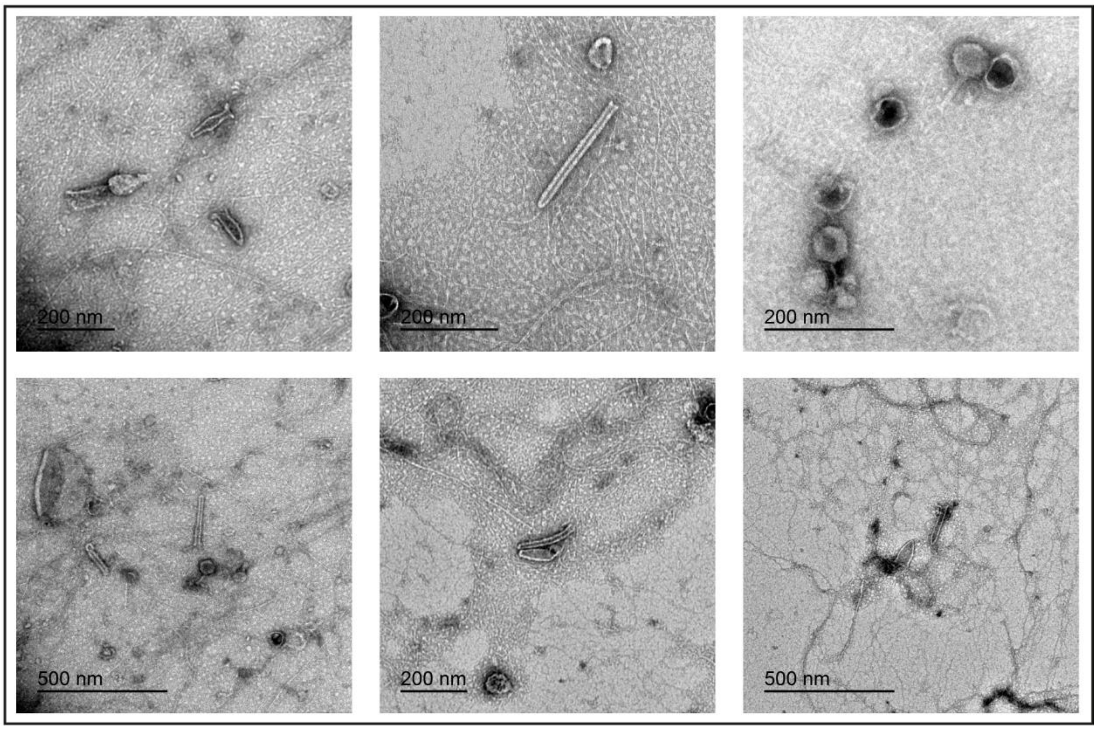

Figure S3. Cell counts assessed with CARD-FISH at the beginning (T0) and the end (Tf) of the experiment, for Bacteria (B) and Archaea (A).

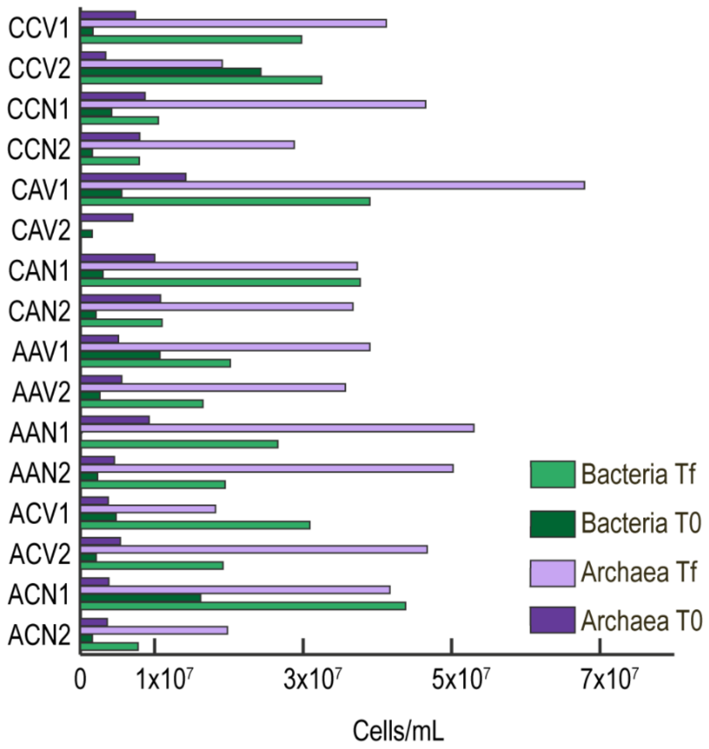

Table S2. Characteristics of analyzed metagenomes from Es Trenc and Aran-Bidgol inoculum brines and from microcosms at the end of the experiment.

Sample Name	Raw lenght (one pair)	Raw reads (one pair)	Trimmed reads (one pair)	% Left after trimming	Nr. coupled reads	Nonpareil coverage	Nr. contigs	N50	Nr. contigs (>500 pb)	Nr. genes predicted (>500 bp)	% Reads mapped to contigs (>500 bp)	% Reads mapped to MAGs**	Nr. High-quality binned MAGs	Genome equivalents	% Community represented by MAG genomospecies
**CZ**	101	25,440,057	22,352,687	88	44,705,374	0.9	81,988	1,702	42,752	102,425	79.14	36.50	13	1279.0	89.72
**CCV1**	151	23,036,876	18,108,620	79	36,217,240	0.9	178,985	1,004	62,101	134,473	82.24	7.24	4	1433.0	91.30
**CCV2**	101	29,400,083	22,942,679	78	45,885,358	0.9	178,985	1,567	46,523	97,785	77.78	50.52	8	1246.9	87.33
**CCN1**	101	37,766,963	29,214,105	77	58,428,210	0.9	114,545	1,429	58,474	119,115	76.73	54.33	12	1510.1	88.92
**CCN2**	151	23,441,348	20,080,935	86	40,161,870	1.0	210,399	870	71,127	150,557	82.03	4.20	3	1511.9	93.74
**CAV1**	101	27,650,298	22,195,294	80	44,390,588	0.9	88,101	1,640	44,559	98,540	76.18	56.32	9	1181.2	90.31
**CAV2**	151	20,794,792	16,857,101	81	33,714,202	0.9	180,682	934	61,958	132,086	80.36	3.61	2	1216.4	99.18
**CAN1**	101	27,586,108	22,514,384	82	45,028,768	0.9	100,388	1,460	51,183	87,000	74.18	49.24	10	1091.1	86.37
**CAN2**	151	26,827,677	22,859,610	85	45,719,220	0.9	234,590	899	82,541	173,038	80.62	6.37	5	1713.6	93.86
**AZ**	101	31,066,351	27,511,857	89	55,023,714	0.7	224,433	863	96,528	199,358	44.73	8.48	9	1456.5	40.14
**AAV1**	101	9,149,241	6,618,705	72	13,237,410	0.5	65,620	787	29,685	30,523	34.12	11.41	2	312.7	44.89
**AAV2**	151	18,020,806	13,528,557	75	27,057,114	0.7	333,070	698	132,297	233,734	60.77	0.85	2	954.8	43.86
**AAN1**	101	5,326,401	3,829,299	72	7,658,598	0.5	34,182	831	14,287	18,634	31.61	12.16	2	165.9	48.18
**AAN2**	151	22,585,243	17,366,621	77	34,733,242	0.8	401,314	736	167,037	299,958	63.79	5.55	2	1167.6	40.01
**ACV1**	101	6,677,239	4,696,719	70	9,393,438	0.4	44,213	674	17,981	16,805	23.02	0.00	0	225.6	37.39
**ACV2**	151	27,299,754	20,227,204	74	40,454,408	0.7	473,227	781	203,410	360,744	60.29	1.55	1	1204.9	34.88
**ACN1**	151	27,223,822	19,377,477	71	38,754,954	0.8	364,781	851	158,532	303,847	66.53	12.92	3	1215.1	32.27
**ACN2**	101	7,766,806	5,560,070	72	11,120,140	0.5	54,558	849	24,419	29,753	36.07	11.68	2	254.6	38.57
**AZV**	101	22,402,143	19,691,076	88	39,382,152	0.7	166,582	755	72,364	177,383	79.57				

* Contig size = 10.000 bp

** Metagenome reads mapped to MAGs binned in the given metagenome.

Table S3. MASH distance between metagenomics reads of inoculum brines and microcosms at the end of the experiment in Es Trenc and Aran-Bidgol samples.

Excel dataset Table S3

Table S4. Statistics of MAGs recovered from metagenomes from Es Trenc and Aran-Bidgol brines and microcosmos samples and MAGs abundances in metagenomes expressed as percentage of metagenome reads recruited to MAGs and normalized by the total of reads in each metagenome and the total MAG genome size (Mb).

Excel dataset Table S4

Table S5. Reciprocal ANI of MAGs comprising genomospecies found in both Es Trenc and Aran-Bidgol metagenomes.

Salinibacter ruber

MAG	A1	Af9	C6	Cf1	Cf23	Cf31	Cf46	Cf51	Cf8
A1	100	98.38	99.43	99.48	99.43	99.41	99.35	99.47	99.38
Af9	98.38	100	98.37	98.15	98	98.52	97.87	97.99	97.88
C6	99.43	98.37	100	99.8	99.75	99.71	99.62	99.73	99.74
Cf1	99.48	98.15	99.8	100	99.77	99.74	99.68	99.75	99.77
Cf23	99.43	98	99.75	99.77	100	99.67	99.59	99.71	99.68
Cf31	99.41	98.52	99.71	99.74	99.67	100	99.55	99.7	99.66
Cf46	99.35	97.87	99.62	99.68	99.59	99.55	100	99.61	99.57
Cf51	99.47	97.99	99.73	99.75	99.71	99.7	99.61	100	99.7
Cf8	99.38	97.88	99.74	99.77	99.68	99.66	99.57	99.7	100

Haloquadratum walsbyi

MAG	A9	Af1	Af4	C5	Cf14	Cf29	Cf40	Cf6
A9	100	99.59	99.66	99.51	99.53	99.52	99.47	99.53
Af1	99.59	100	99.64	99.5	99.53	99.52	99.49	99.53
Af4	99.66	99.64	100	99.54	99.53	99.52	99.5	99.55
C5	99.51	99.5	99.54	100	99.86	99.86	99.82	99.84
Cf14	99.53	99.53	99.53	99.86	100	99.87	99.83	99.87
Cf29	99.52	99.52	99.52	99.86	99.87	100	99.83	99.86
Cf40	99.47	99.49	99.5	99.82	99.83	99.83	100	99.84
Cf6	99.53	99.53	99.55	99.84	99.87	99.86	99.84	100

Halonotius sp.

MAG	Cf47
Af8	98.9

Table S6. Bray Curtis dissimilarity between inoculum brines and microcosms at the end of the experiment in Es Trenc and Aran-Bidgol samples based on relative abundances of MAGs.

	CZ	CCV1	CCV2	CCN1	CCN2	CAV1	CAV2	CAN1	CAN2
**CZ**	0.000	0.162	0.087	0.104	0.175	0.154	0.242	0.148	0.230
**CCV1**	0.162	0.000	0.081	0.108	0.058	0.048	0.082	0.107	0.082
**CCV2**	0.087	0.081	0.000	0.054	0.089	0.079	0.161	0.091	0.147
**CCN1**	0.104	0.108	0.054	0.000	0.101	0.105	0.188	0.076	0.164
**CCN2**	0.175	0.058	0.089	0.101	0.000	0.065	0.094	0.076	0.063
**CAV1**	0.154	0.048	0.079	0.105	0.065	0.000	0.091	0.073	0.090
**CAV2**	0.242	0.082	0.161	0.188	0.094	0.091	0.000	0.132	0.050
**CAN1**	0.148	0.107	0.091	0.076	0.076	0.073	0.132	0.000	0.099
**CAN2**	0.230	0.082	0.147	0.164	0.063	0.090	0.050	0.099	0.000

	AZ	ACV1	ACV2	ACN1	ACN2	AAV1	AAV2	AAN1	AAN2
**AZ**	0.000	0.207	0.202	0.560	0.564	0.203	0.238	0.360	0.288
**ACV1**	0.207	0.000	0.170	0.577	0.555	0.115	0.127	0.245	0.276
**ACV2**	0.202	0.170	0.000	0.530	0.537	0.253	0.226	0.364	0.273
**ACN1**	0.560	0.577	0.530	0.000	0.174	0.562	0.567	0.326	0.307
**ACN2**	0.564	0.555	0.537	0.174	0.000	0.522	0.569	0.340	0.369
**AAV1**	0.203	0.115	0.253	0.562	0.522	0.000	0.086	0.192	0.283
**AAV2**	0.238	0.127	0.226	0.567	0.569	0.086	0.000	0.206	0.261
**AAN1**	0.360	0.245	0.364	0.326	0.340	0.192	0.206	0.000	0.140
**AAN2**	0.288	0.276	0.273	0.307	0.369	0.283	0.261	0.140	0.000

Table S7. Statistics of viral genomes recovered from the metavirome from Aran-Bidgol inoculum, viral abundances in metagenomes expressed as percentage of metagenome reads recruited to viral genomes and normalized by the total of reads in each metagenome and the total viral genome size (Mb), ANIr values in each metagenome, putative hosts and number of integrases. Only viral contigs with sequencing breadth > 70 are shown.

Excel dataset Table S7

Table S8. Statistics of viral genomes recovered from metagenomes from Es Trenc and Aran-Bidgol brines and microcosms, viral abundances in metagenomes expressed as percentage of metagenome reads recruited to viral genomes and normalized by the total of reads in each metagenome and the total viral genome size (Mb), ANIr values in each metagenome, putative hosts and number of integrases. Only viral contigs with sequencing breadth > 70 are shown.

Excel dataset Table S8

Figure S4. ViPTree proteome-based tree of viral genomes retrieved from cellular metagenomes.

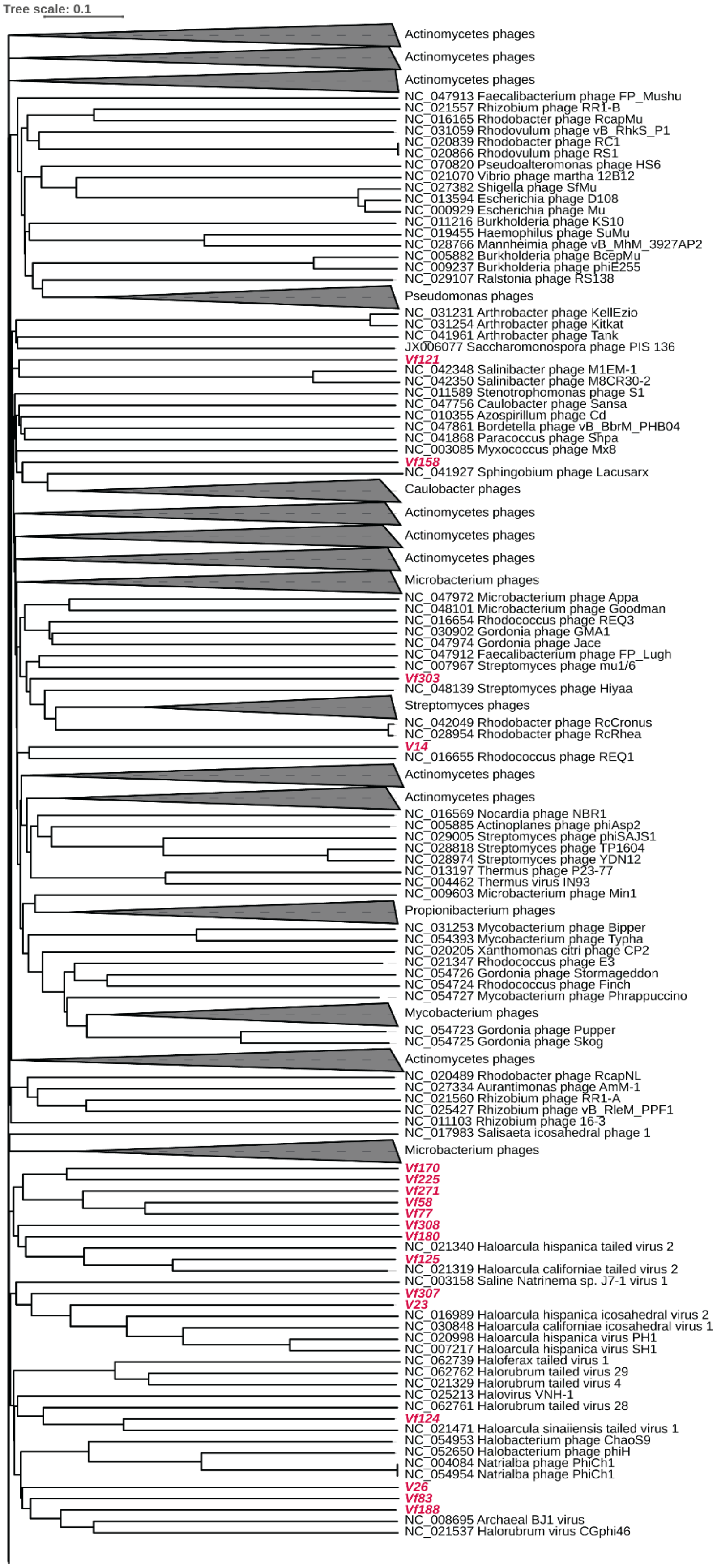

Cont.

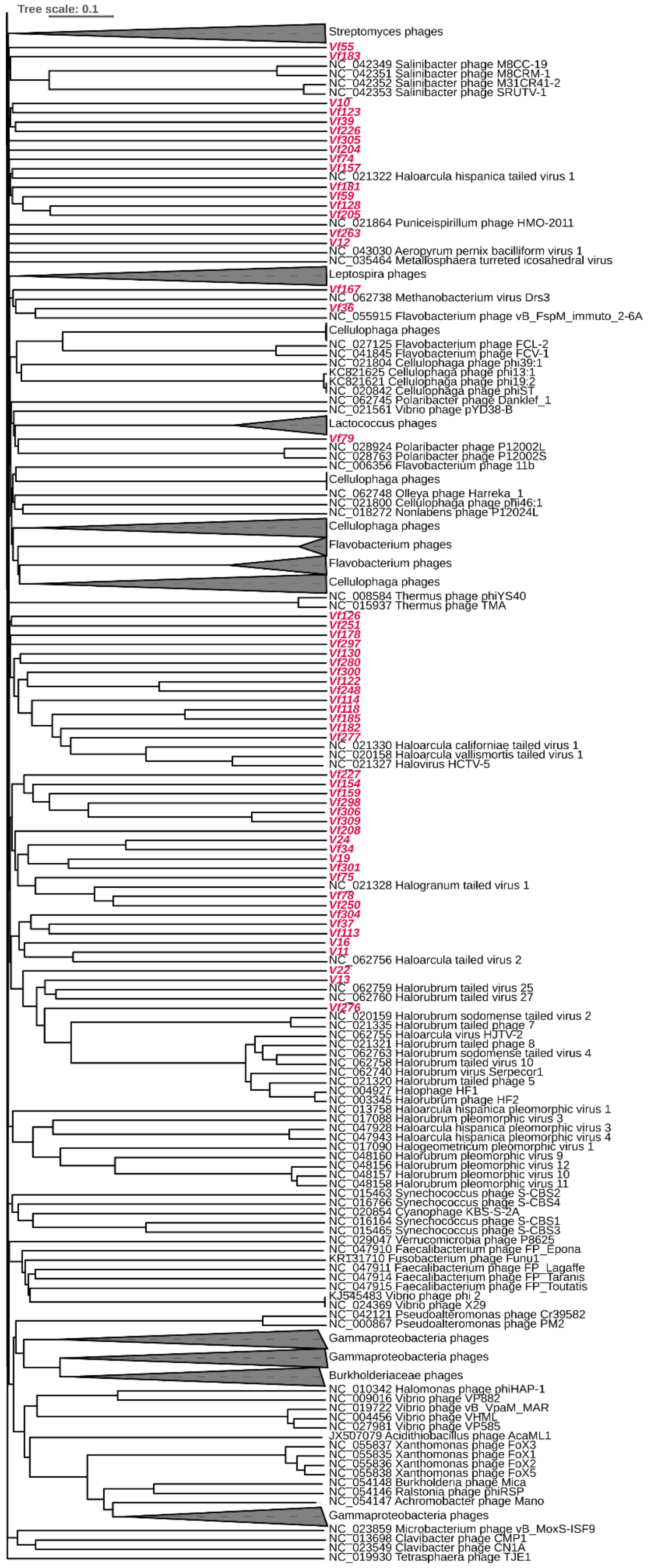

Tree scale: 0.1
Streptomyces phages
Vf55
Vf183
NC_042349 Salinibacter phage M8CC-19
NC_042351 Salinibacter phage M8CRM-1
NC_042352 Salinibacter phage M31CR41-2
NC_042353 Salinibacter phage SRUTV-1
V10
Vf123
Vf39
Vf226
Vf305
Vf204
Vf74
Vf157
NC_021322 Haloarcula hispanica tailed virus 1
Vf181
Vf59
Vf128
Vf205
NC_021864 Puniceispirillum phage HMO-2011
Vf263
V12
NC_043030 Aeropyrum pernix bacilliform virus 1
NC_035464 Metallosphaera turreted icosahedral virus
Leptospira phages
Vf167
NC_062738 Methanobacterium virus Drs3
Vf36
NC_055915 Flavobacterium phage vB_FspM_immuto_2-6A
Cellulophaga phages
NC_027125 Flavobacterium phage FCL-2
NC_041845 Flavobacterium phage FCV-1
NC_021804 Cellulophaga phage phi39:1
KC821625 Cellulophaga phage phi13:1
KC821621 Cellulophaga phage phi19:2
NC_020842 Cellulophaga phage phiST
NC_062745 Polaribacter phage Danklef_1
NC_021561 Vibrio phage pYD38-B
Lactococcus phages
Vf79
NC_028924 Polaribacter phage P12002L
NC_028763 Polaribacter phage P12002S
NC_006356 Flavobacterium phage 11b
Cellulophaga phages
NC_062748 Olleya phage Harreka_1
NC_021800 Cellulophaga phage phi46:1
NC_018272 Nonlabens phage P12024L
Cellulophaga phages
Flavobacterium phages
Flavobacterium phages
Cellulophaga phages
NC_008584 Thermus phage phiYS40
NC_015937 Thermus phage TMA
Vf126
Vf251
Vf178
Vf297
Vf130
Vf280
Vf300
Vf122
Vf248
Vf114
Vf118
Vf185
Vf182
Vf277
NC_021330 Haloarcula californiae tailed virus 1
NC_020158 Haloarcula vallismortis tailed virus 1
NC_021327 Halovirus HCTV-5
Vf227
Vf154
Vf159
Vf298
Vf306
Vf309
Vf208
V24
Vf34
V19
Vf301
Vf75
NC_021328 Halogranum tailed virus 1
Vf78
Vf250
Vf304
Vf37
Vf113
V16
V11
NC_062756 Haloarcula tailed virus 2
V22
V13
NC_062759 Halorubrum tailed virus 25
NC_062760 Halorubrum tailed virus 27
Vf276
NC_020159 Halorubrum sodomense tailed virus 2
NC_021335 Halorubrum tailed phage 7
NC_062755 Haloarcula virus HJTV-2
NC_021321 Halorubrum tailed phage 8
NC_062763 Halorubrum sodomense tailed virus 4
NC_062758 Halorubrum tailed virus 10
NC_062740 Halorubrum virus Serpecor1
NC_021320 Halorubrum tailed phage 5
NC_004927 Halophage HF1
NC_003345 Halorubrum phage HF2
NC_013758 Haloarcula hispanica pleomorphic virus 1
NC_017088 Halorubrum pleomorphic virus 3
NC_047928 Haloarcula hispanica pleomorphic virus 3
NC_047943 Haloarcula hispanica pleomorphic virus 4
NC_017090 Halogeometricum pleomorphic virus 1
NC_048160 Halorubrum pleomorphic virus 9
NC_048156 Halorubrum pleomorphic virus 12
NC_048157 Halorubrum pleomorphic virus 10
NC_048158 Halorubrum pleomorphic virus 11
NC_015463 Synechococcus phage S-CBS2
NC_016766 Synechococcus phage S-CBS4
NC_020854 Cyanophage KBS-S-2A
NC_016164 Synechococcus phage S-CBS1
NC_015465 Synechococcus phage S-CBS3
NC_029047 Verrucomicrobia phage P8625
NC_047910 Faecalibacterium phage FP_Epona
KR131710 Fusobacterium phage Funu1
NC_047911 Faecalibacterium phage FP_Lagaffe
NC_047914 Faecalibacterium phage FP_Taranis
NC_047915 Faecalibacterium phage FP_Toutatis
KJ545483 Vibrio phage phi 2
NC_024369 Vibrio phage X29
NC_042121 Pseudoalteromonas phage Cr39582
NC_000867 Pseudoalteromonas phage PM2
Gammaproteobacteria phages
Gammaproteobacteria phages
Burkholderiaceae phages
NC_010342 Halomonas phage phiHAP-1
NC_009016 Vibrio phage VP882
NC_019722 Vibrio phage vB_VpaM_MAR
NC_004456 Vibrio phage VHML
NC_027981 Vibrio phage VP585
JX507079 Acidithiobacillus phage AcaML1
NC_055837 Xanthomonas phage FoX3
NC_055835 Xanthomonas phage FoX1
NC_055836 Xanthomonas phage FoX2
NC_055838 Xanthomonas phage FoX5
NC_054148 Burkholderia phage Mica
NC_054146 Ralstonia phage phiRSP
NC_054147 Achromobacter phage Mano
Gammaproteobacteria phages
NC_023859 Microbacterium phage vB_MoxS-ISF9
NC_013698 Clavibacter phage CMP1
NC_023549 Clavibacter phage CN1A
NC_019930 Tetrasphaera phage TJE1

Figure S5. db-RDA ordination of Es Trenc MAGs based on the Bray Curtis distance of the sequencing depth of MAG contig genome windows (n=1.000) in each metagenome, normalized by the MAG's sequencing depth. The experimental conditions (origin of the cells and brines and the presence/absence of viruses) were used as constraining variables. The relative contribution (eigenvalue) of each axis to the total inertia in the data as well as to the constrained space only, respectively, are indicated in percent at the axis titles.

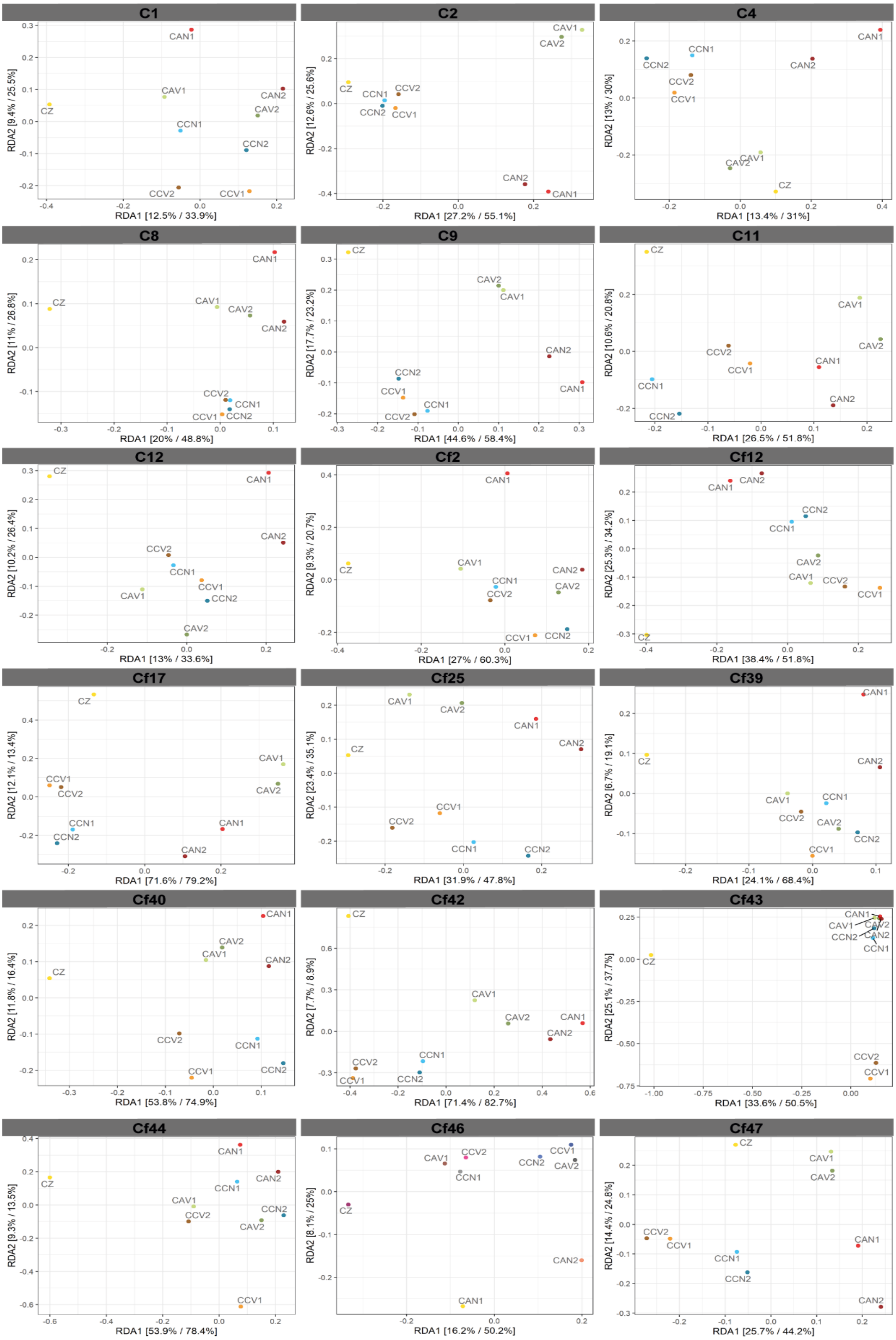
C1
RDA1 [12.5% / 33.9%]
RDA2 [9.4% / 25.5%]
C2
RDA1 [27.2% / 55.1%]
RDA2 [12.6% / 25.6%]
C4
RDA1 [13.4% / 31%]
RDA2 [13% / 30%]
C8
RDA1 [20% / 48.8%]
RDA2 [11% / 26.8%]
C9
RDA1 [44.6% / 58.4%]
RDA2 [17.7% / 23.2%]
C11
RDA1 [26.5% / 51.8%]
RDA2 [10.6% / 20.8%]
C12
RDA1 [13% / 33.6%]
RDA2 [10.2% / 26.4%]
Cf2
RDA1 [27% / 60.3%]
RDA2 [9.3% / 20.7%]
Cf12
RDA1 [38.4% / 51.8%]
RDA2 [25.3% / 34.2%]
Cf17
RDA1 [71.6% / 79.2%]
RDA2 [12.1% / 13.4%]
Cf25
RDA1 [31.9% / 47.8%]
RDA2 [23.4% / 35.1%]
Cf39
RDA1 [24.1% / 68.4%]
RDA2 [6.7% / 19.1%]
Cf40
RDA1 [53.8% / 74.9%]
RDA2 [11.8% / 16.4%]
Cf42
RDA1 [71.4% / 82.7%]
RDA2 [7.7% / 8.9%]
Cf43
RDA1 [33.6% / 50.5%]
RDA2 [25.1% / 37.7%]
Cf44
RDA1 [53.9% / 78.4%]
RDA2 [9.3% / 13.5%]
Cf46
RDA1 [16.2% / 50.2%]
RDA2 [8.1% / 25%]
Cf47
RDA1 [25.7% / 44.2%]
RDA2 [14.4% / 24.8%]
CZ
CAV1
CAV2
CCV1
CCV2
CCN1
CCN2
CAN1
CAN2

Figure S6. db-RDA ordination of Es Trenc MAGs based on the Bray Curtis distance of the sequencing depth of MAG contig genome windows (n=1.000) in each metagenome, normalized by the MAG's sequencing depth. The experimental conditions (origin of the cells and brines and the presence/absence of viruses) were used as constraining variables. The relative contribution (eigenvalue) of each axis to the total inertia in the data as well as to the constrained space only, respectively, are indicated in percent at the axis titles.

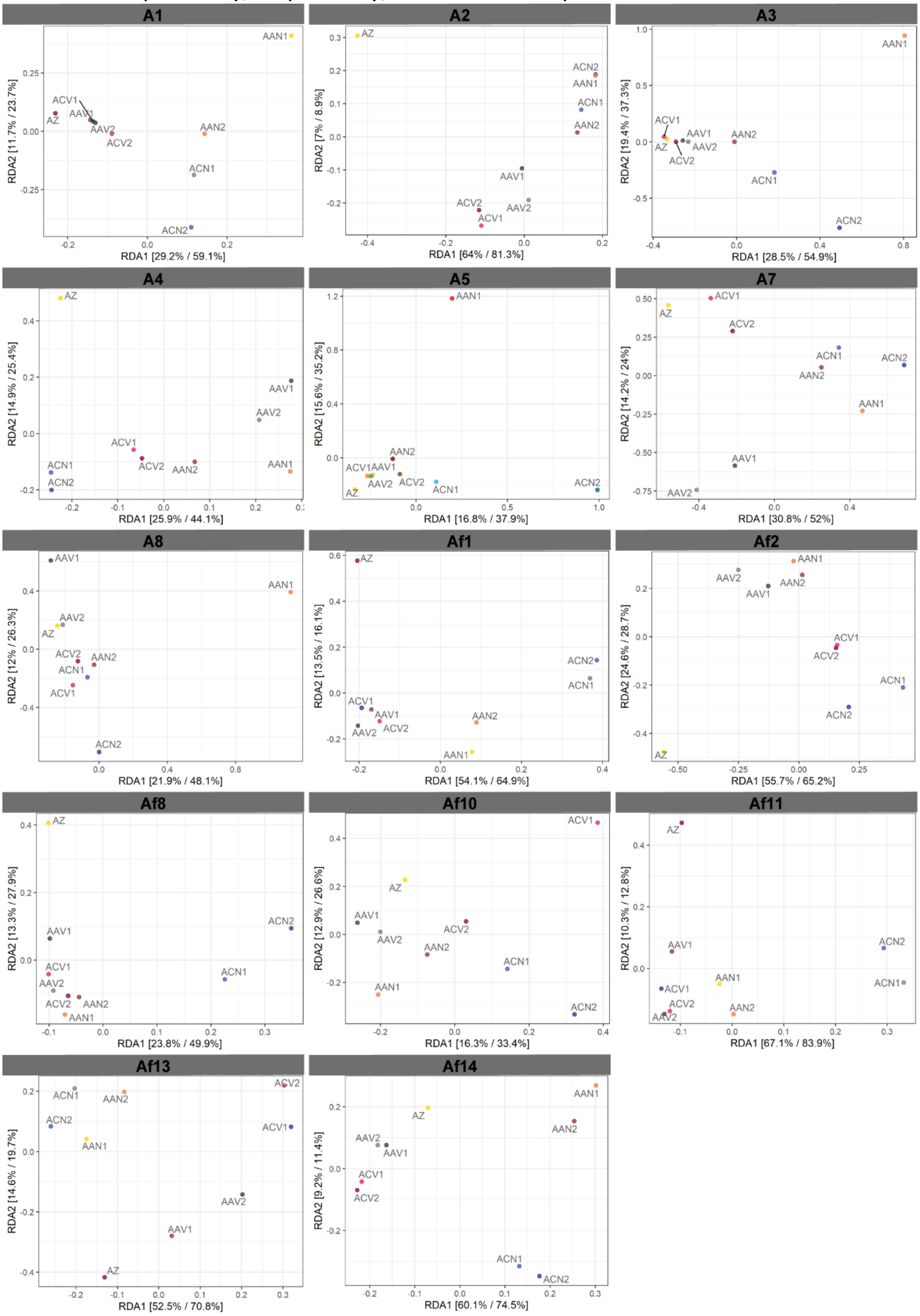

Figure S7. Selected plots of metagenome reads recruited to MAGs contigs in Aran-Bidgol and Es Trenc.

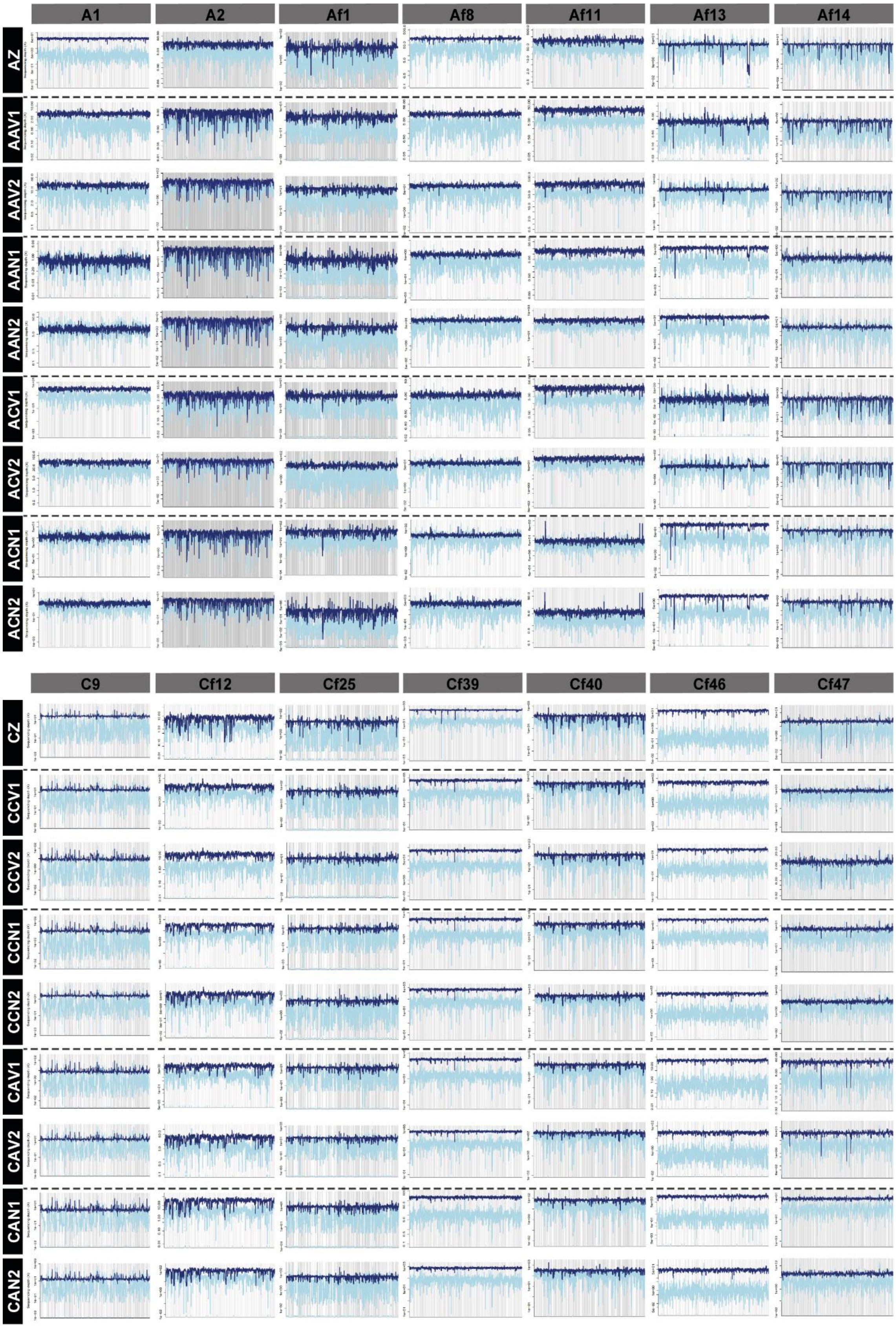

Table S9. Log2-fold gene abundance differences of selected MAGs between microcosms due to the change of environment and the presence/absence of autochthonous/allochthonous viruses. Genes with log fold change >2 and p <0.05 are displayed as percentage of total genes. The number of genes related with infection significantly higher in a given condition is shown.

Excel dataset Table S9

Figure S8. Specific genes of MAGs Af1 and Cf40 grouped by gene category. Genes are shown in percentage of genes in a given category relative to the total specific genes in the particular MAG. Non annotated genes added up to 57% in MAG Af1 and 73% in MAG Cf40.

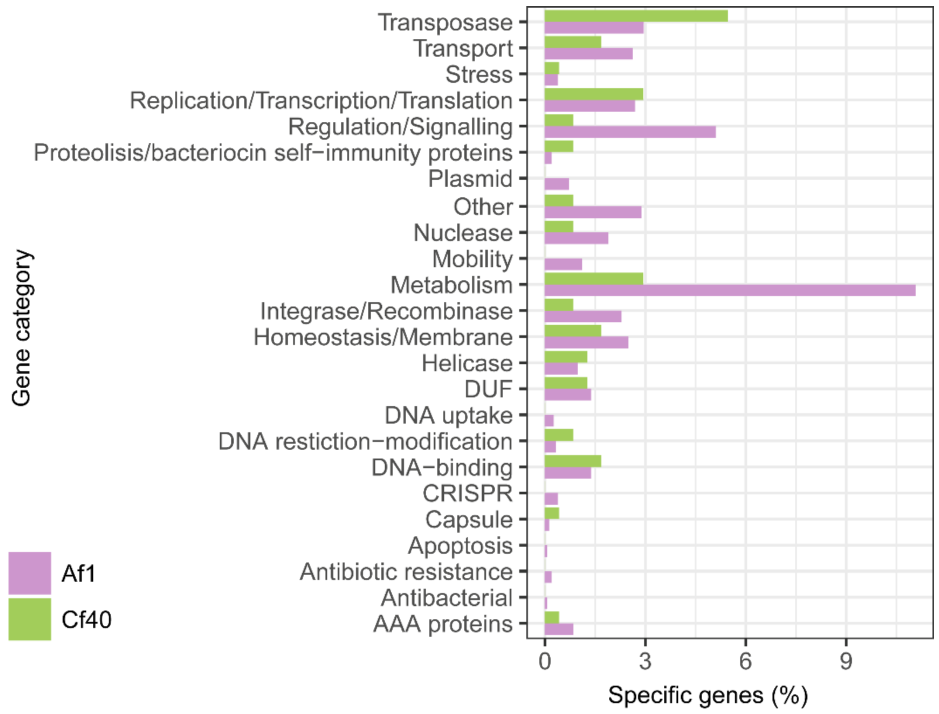

Figure S9. Plot of metagenome reads recruited to MAGs contigs of genomospecies *Salinibacter ruber* **(A)** and *Halohasta* sp. **(H)** in Aran-Bidgol and Es Trenc; RDA (redundancy analysis) of MAGs of genomospecies *Salinibacter ruber* **(D, E)** and *Halohasta* sp. **(I, J)** in Aran-Bidgol and Es Trenc respectively based on Bray Curtis distance and constrained by the experimental condition of the sequencing depth of contig genome windows (n=1.000) in each metagenome; MA-plot showing the $\log_2$ fold changes of gene counts over the $\log_2$ mean of genomospecies *Salinibacter ruber* **(D-G)** and *Halohasta* sp. **(K-N)** comparing microcosms with presence/absence of autochthonous (D, E, K, L) and allochthonous (F, G, M, N) viruses. Genes with statistically significant ($p<0.05$) $\log_2$ fold changes > 2 are displayed in red and $\log_2$ fold changes < -2 in blue.

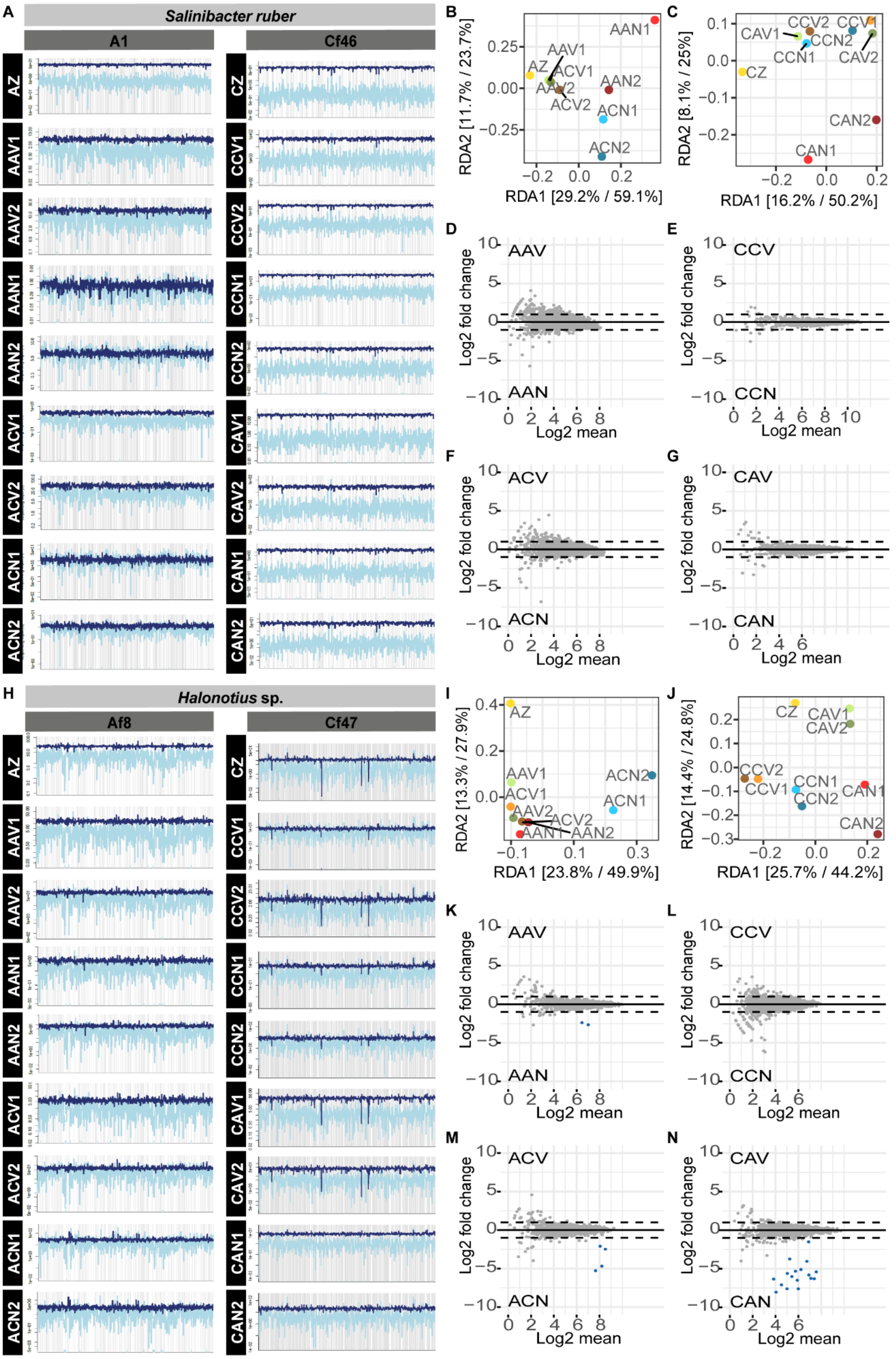
A
Salinibacter ruber
A1
Cf46
AZ
AAV1
AAV2
AAN1
AAN2
ACV1
ACV2
ACN1
ACN2
CZ
CCV1
CCV2
CCN1
CCN2
CAV1
CAV2
CAN1
CAN2
B
RDA2 [11.7% / 23.7%]
RDA1 [29.2% / 59.1%]
C
RDA2 [8.1% / 25%]
RDA1 [16.2% / 50.2%]
D
AAV
AAN
Log2 fold change
Log2 mean
E
CCV
CCN
F
ACV
ACN
G
CAV
CAN
H
Halonotius sp.
Af8
Cf47
I
RDA2 [13.3% / 27.9%]
RDA1 [23.8% / 49.9%]
J
RDA2 [14.4% / 24.8%]
RDA1 [25.7% / 44.2%]
K
L
M
N

Table S10. Pairwise ANIr differences of MAGs in Es Trenc and Aran-Bidgol samples between inoculum brines and microcosms at the end of the experiment and between microcosms with and without suspended viruses in brine. Shown in red when ANIr of MAGs increased at the end of the experiment compared to the inoculum, and in blue, when ANIr of MAGs decreased at the end of the experiment compared to the inoculum. Only MAGs with a sequencing breath higher than 70 in a given metagenome were considered.

MAG	CCV1-CZ	CCV2-CZ	CCN1-CZ	CCN2-CZ	CAV1-CZ	CAV2-CZ	CAN1-CZ	CAN2-CZ
**C1**	-0.10	-0.03	-0.03	-0.10	-0.04	-0.10	-0.04	-0.11
**C2**	-0.08	-0.12	-0.11	-0.06	-0.35	-0.20	-0.23	-0.16
**C4**	-0.32	-0.18	-0.16	-0.30	-0.17	-0.31	-0.18	-0.31
**C8**	-0.05	0.01	0.03	-0.02	0.00	-0.06	0.02	-0.02
**C9**	-0.10	-0.12	-0.07	-0.04	-0.20	-0.16	-0.22	-0.16
**C11**	-0.13	-0.04	-0.07	-0.13	-0.04	-0.13	-0.06	-0.13
**C12**	-0.03	-0.04	-0.05	-0.03	-0.06	-0.04	-0.07	-0.04
**Cf2**	-0.04	0.01	0.01	-0.02	0.01	-0.03	0.00	-0.01
**Cf12**	0.13	0.42	0.28	0.09	0.20	0.06	0.25	0.09
**Cf17**	0.04	0.04	0.08	0.04	-0.06	-0.10	-0.04	-0.04
**Cf25**	0.06	-0.02	0.01	0.10	0.01	0.07	0.02	0.08
**Cf39**	-0.06	0.01	0.00	-0.06	0.01	-0.06	0.00	-0.06
**Cf40**	0.00	0.04	0.05	0.00	0.01	-0.02	0.03	0.00
**Cf42**								
**Cf43**								
**Cf44**	-0.12	0.04	0.11	-0.04	0.00	-0.20	0.20	-0.04
**Cf46**	-0.10	0.01	0.01	-0.09	0.01	-0.10	0.05	-0.11
**Cf47**	-0.11	0.00	0.01	-0.10	0.09	-0.04	0.20	-0.05

MAG	AAV1-AZ	AAV2-AZ	AAN1-AZ	AAN2-AZ	ACV1-AZ	ACV2-AZ	ACN1-AZ	ACN2-AZ
**A1**	-0.15	-0.19	-0.54	-0.61	-0.21	-0.31	-0.61	-0.45
**A2**	-0.16	-0.33	-0.18	-0.36	-0.16	-0.34	-0.35	-0.16
**A3**	-0.05	-0.10		-0.20	-0.01	-0.08	-0.23	-0.22
**A4**	0.08	0.11	0.08	0.11	0.07	0.09	0.10	0.09
**A5**	-0.21	-0.11	-0.52	-0.08	-0.30	-0.50	-0.29	-0.84
**A7**	-0.73			-0.59	-0.57	-0.50	-0.52	
**A8**	-0.05	-0.05	-0.10	-0.03	0.03	-0.02	-0.02	-0.02
**Af1**	0.12	0.09	0.10	0.10	0.08	0.15	0.15	0.16
**Af2**	0.10	0.00	0.12	0.14	0.26	0.22	0.48	0.32
**Af8**	0.08	-0.11	-0.04	-0.10	-0.06	-0.15	-0.14	-0.07
**Af10**	0.11	0.17	0.04	0.07	-0.07	0.03	0.00	-0.08
**Af11**	0.08	-0.14	0.22	-0.12	0.08	-0.15	-0.10	0.10
**Af13**	0.18	0.15	0.26	0.23	0.12	0.14	0.25	0.29
**Af14**	0.04	0.01	-0.05	-0.03	0.03	-0.01	-0.04	-0.03
